# ZeoSyn: A Comprehensive Zeolite Synthesis Dataset Enabling Machine-Learning Rationalization of Hydrothermal Parameters

**DOI:** 10.1021/acscentsci.3c01615

**Published:** 2024-03-06

**Authors:** Elton Pan, Soonhyoung Kwon, Zach Jensen, Mingrou Xie, Rafael Gómez-Bombarelli, Manuel Moliner, Yuriy Román-Leshkov, Elsa Olivetti

**Affiliations:** † Department of Materials Science and Engineering, Massachusetts Institute of Technology, Cambridge, Massachusetts 02139, United States; ‡ Department of Chemical Engineering, Massachusetts Institute of Technology, Cambridge, Massachusetts 02139, United States; ¶ Instituto de Tecnología Química, Universitat Politècnica de València-Consejo Superior de Investigaciones Científicas 46022, Valencia, Spain

## Abstract

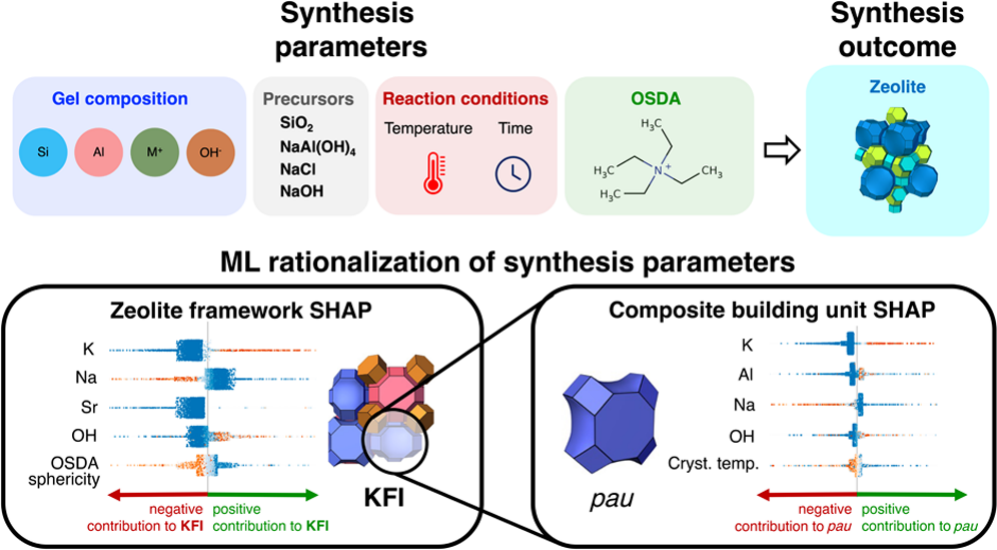

Zeolites, nanoporous aluminosilicates with well-defined porous structures, are versatile materials with applications in catalysis, gas separation, and ion exchange. Hydrothermal synthesis is widely used for zeolite production, offering control over composition, crystallinity, and pore size. However, the intricate interplay of synthesis parameters necessitates a comprehensive understanding of synthesis–structure relationships to optimize the synthesis process. Hitherto, public zeolite synthesis databases only contain a subset of parameters and are small in scale, comprising up to a few thousand synthesis routes. We present ZeoSyn, a dataset of 23,961 zeolite hydrothermal synthesis routes, encompassing 233 zeolite topologies and 921 organic structure-directing agents (OSDAs). Each synthesis route comprises comprehensive synthesis parameters: 1) gel composition, 2) reaction conditions, 3) OSDAs, and 4) zeolite products. Using ZeoSyn, we develop a machine learning classifier to predict the resultant zeolite given a synthesis route with >70% accuracy. We employ SHapley Additive exPlanations (SHAP) to uncover key synthesis parameters for >200 zeolite frameworks. We introduce an aggregation approach to extend SHAP to all building units. We demonstrate applications of this approach to phase-selective and intergrowth synthesis. This comprehensive analysis illuminates the synthesis parameters pivotal in driving zeolite crystallization, offering the potential to guide the synthesis of desired zeolites. The dataset is available at https://github.com/eltonpan/zeosyn_dataset.

## Abstract 2

Zeolites are nanoporous materials with applications in catalysis and gas separation. We open-source the largest dataset on zeolite synthesis and develop an interpretable machine learning framework to reveal key synthesis−structure relationships.

## Introduction

Zeolites are nanoporous, crystalline aluminosilicate materials with a wide range of industrial applications including catalysis, separations, and ion exchange.^1−3^ In addition to composition, the crystalline structure and corresponding porous network are crucial in determining a zeolite’s suitability for a target application.^4,5^ While thousands of potential zeolite structures are thought to be thermodynamically accessible,^6^ only 264 have been synthesized^7^ highlighting a synthesis bottleneck to zeolite discovery and deployment. Zeolite synthesis has typically been based on trial-and-error methods guided by accumulated domain knowledge.^8^ The synthesis of zeolites is intricate, with numerous variables influencing the resultant zeolite structure.^9^ These factors include framework heteroatoms, the presence of inorganic and organic cations, structure-directing agents, mineralizing agents, and hydrothermal conditions.^1,8−11^

Many studies have examined parts of the zeolite synthesis space including compositional gel ratios (Si/Al, Na^+^/Si, OSDA/Si, H_2_O/Si, etc.),^12−16^ aging conditions,^17−19^ crystallization conditions,^20−22^ and precursor selection^23−25^ for specific OSDAs.^5^ However, knowledge of the holistic interplay between these factors across the entire field is lacking. Data science and machine learning have shown promise in generalizing some synthesis–structure relationships^26−29^ but have been limited to subsections of the zeolite design space due to a lack of data, which implies that larger datasets may generalize learning more broadly across the zeolite space.

Previous works have curated zeolite synthesis datasets. Specifically, a dataset consisting of 1,200 unique synthetic routes for Ge-containing zeolites has been reported by Jensen et al.^28^ In the same vein, Yan et al. compiled a database of 1,600 synthetic records of open-framework aluminophosphate (AlPO) syntheses.^30^ However, these datasets cover a subset of frameworks, giving rise to the first issue of *data scarcity*. There have been datasets that integrate synthesis information across the field of zeolites. For instance, Schwalbe-Koda et al. leveraged atomistic simulations to calculate the binding energies and OSDA features for more than 100,000 zeolite–OSDA pairs.^31^ Moreover, Jensen et al. curated a dataset of OSDAs used in >5,000 synthesis routes.^32^ However, these datasets contain only a subset of key parameters (containing only gel composition, OSDA, or reaction conditions, but not all three), giving rise to the second issue of *data sparsity*. Muraoka et al.^29^ curated a comprehensive dataset comprising gel compositions and reaction conditions but without OSDAs, a key component of zeolite synthesis. This dataset (Muraoka et al.) contains 686 unique synthesis routes for 23 unique frameworks, covering only 9% of synthesized frameworks. A more complete dataset would address the scarcity and sparsity issues limiting generalized data-driven learning opportunities.

Within the published literature, synthesis recipes of zeolites are commonly reported in the experimental and supporting information sections in the form of text and tables. Data-mining using natural language processing (NLP) frameworks have been developed to extract zeolite synthesis data.^28,32−35^ Given the need for a highly curated dataset involving multiple synthesis parameters (ranging from gel composition and reaction conditions to OSDAs), a hybrid approach involving NLP coupled with manual checking ensures high data quality. Thereafter, combining literature-extracted data from the entire zeolite domain with machine learning (ML) modeling^28,32,36^ could expand on understanding of zeolite synthesis.

**Figure 1:**
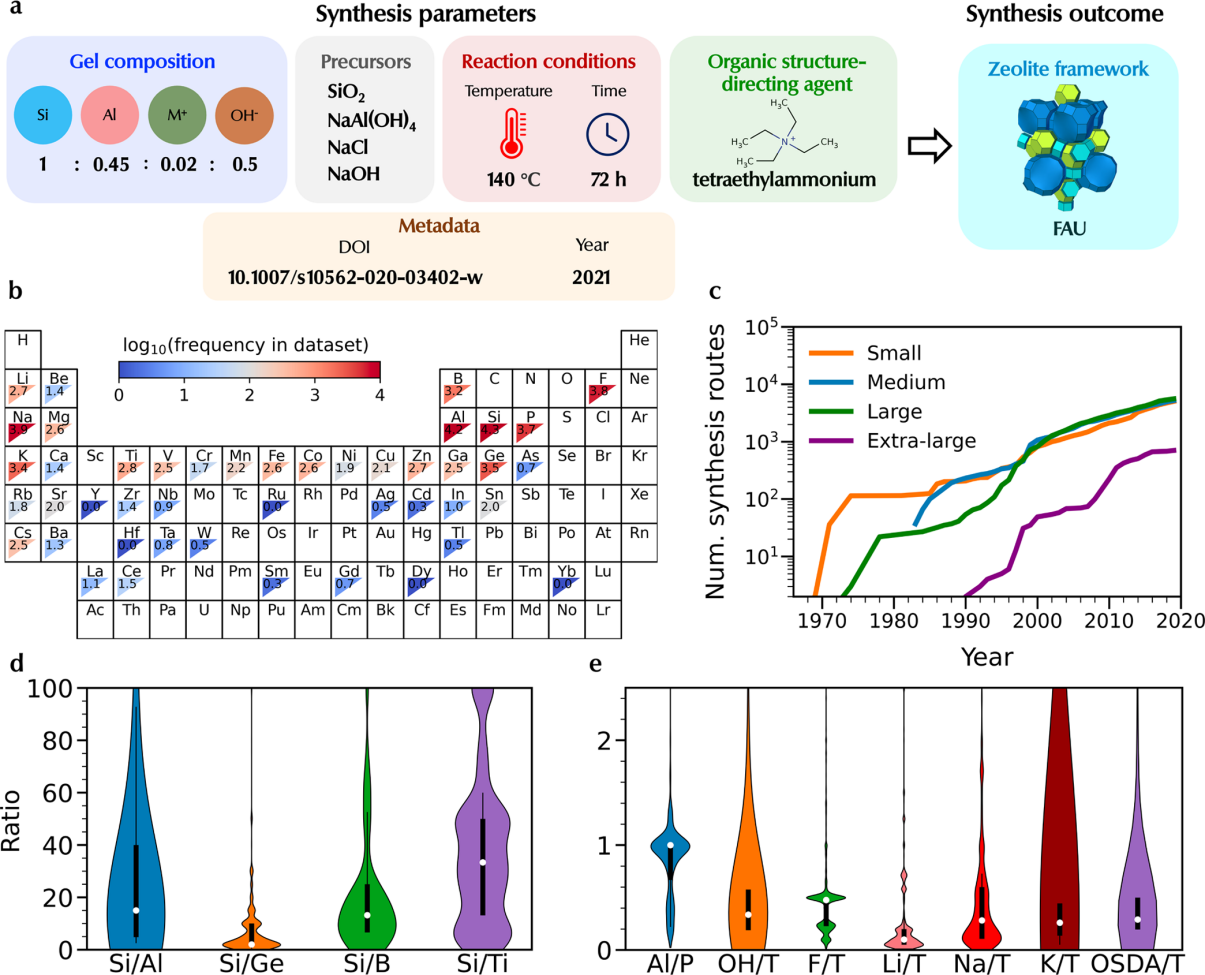
The ZeoSyn dataset. (a) An example of a zeolite synthesis route (1 out of 23,961) in the dataset, consisting of the gel composition, inorganic precursors, reaction conditions, organic structure-directing agent (OSDA), and the resultant zeolite framework. Paper metadata of the scientific paper containing the synthesis route is also provided. (b) Frequency of elements present in the dataset. The values correspond to the logarithm of synthetic routes with a specific element. (c) Total number of synthesis routes of small, medium, large, and extra-large pore zeolites extracted from literature across time in the dataset. Distributions of key gel composition variables in the dataset, including ratio between (d) heteroatoms, and (e) mineralizing agents, metal cations, and OSDA ratios (*T* = ∑_*i*_*n*_*i*_ where *n*_*i*_ is the amount of the *i*th heteroatom present in synthesis).

We present ZeoSyn, a comprehensive dataset of 23,961 zeolite synthesis routes for 233 unique zeolite frameworks (covering >80% of synthesized frameworks to date) and 921 unique OSDAs. This dataset is an order of magnitude larger than any previously published datasets on zeolite synthesis. Each unique synthesis route in ZeoSyn is *comprehensive*, consisting of gel composition, reaction conditions, inorganic precursors, OSDAs, and the resulting zeolite structure extracted from the scientific literature (Figure 1a). We examine relationships between hydrothermal variables, OSDAs, and resulting zeolite structures by exploring ZeoSyn to highlight trends within the zeolite synthesis space. We train a supervised classification machine learning model on ZeoSyn to predict zeolite framework products given a synthesis route. We employ SHapley Additive exPlanations (SHAP) to reveal the most important synthesis parameters driving the formation of over 200 zeolite frameworks and their constituent composite building units (CBUs) and show potential applications in phase-selective and intergrowth synthesis. Analysis at this scale is a step toward an improved understanding of key synthesis parameters driving zeolite crystallization, which could potentially guide and accelerate the discovery of new zeolite frameworks.

## Results and Discussion

### Extracted Dataset

The dataset presented in this work contains comprehensive synthesis information on zeolites including gel composition, reaction conditions (crystallization time/temperature), precursors, and OSDAs as shown in Figure 1a. The dataset also includes the resulting zeolite structures formed (or lack thereof, e.g., dense/amorphous phases) for each synthesis route and, in some instances, zeolite properties such as Si/Al ratio in the product, crystal size, percent crystallinity, and BET surface area. The dataset consists of 23,961 synthesis routes from 3,096 journal articles spanning the years 1966–2021. It contains data on 921 unique OSDA molecules, 233 zeolite structures, and 1,022 unique materials. The extracted gel compositions are a combination of 51 different gel components, including Si, Al, P, Na^+^, K^+^, OH^–^, F^–^, Ge, Ti, B, Ga, V, OSDA, H_2_O, and additional solvents. Each unique synthesis route also contains the Digital Object Identifier (DOI) and the year of publication of the scientific paper from which it was extracted.

#### Element Frequencies

The elemental frequencies in Figure 1b show the wide diversity of elements used in zeolite synthesis space, ranging from alkali, alkaline-earth metals, and transition metals to p-block elements. Elements with high frequencies (shown in red) include Si, Al, P, Ge, and B, serving as heteroatoms of the framework. Other common elements, such as Group I metal ions Na^+^ and K^+^, act as inorganic structure-directing agents, while OH^–^ and F^–^ act as mineralizing agents to solubilize Al and Si sources. Group II metal ions, such as Sr^2+^ and Ba^2+^, have been reported to accelerate the crystallization of zeolite frameworks such as **CHA**(37) and **LTL**,^38^ respectively.

Some elements confer structural stability to the framework. Ge confers framework flexibility and high tolerance in the framework structure to acute T–O–T bond angles.^39,40^ Zn also belongs to a group of flexible heteroatoms that can facilitate the formation of 3-membered T atom rings in frameworks.^9,41^ In addition to their structure-directing role in the crystallization of some frameworks, other elements (such as B) have been incorporated into the framework for subsequent Al substitution.^42−44^ P is a fundamental heteroatom for the synthesis of P-based zeotypes, such as aluminophosphates (AlPOs), silicoaluminophosphates (SAPOs), and metalloaluminophosphates (MeAlPOs).^45,46^

Many transition elements have been incorporated into zeolite frameworks to introduce new chemical functionalities beyond Brønsted acid sites for novel catalytic applications. Ti, Sn, and Zr serve as Lewis acid sites for selective oxidation reactions.^47−49^ Fe and Cu serve as extra-framework catalytic sites in zeolites for NO_*x*_ reduction,^50,51^ among other chemical processes. Zeolites have served as supports for transition metal catalysts, like Co and Ni, to obtain fuels from syngas^52^ and CO_2_ methanation.^53^ Lanthanides, such as paramagnetic Gd, have been incorporated in zeolites for biomedical applications such as magnetic resonance imaging.^54^

#### Zeolite Frameworks

Zeolite frameworks can be divided into different categories based on their maximum ring size. ZeoSyn contains 5,250, 5,494, 5,769, and 716 synthesis routes for small (≤8-membered rings), medium (≤10-membered rings), large (≤12-membered rings), and extra-large pore (>12-membered rings) zeolites, respectively. The most common zeolite in the dataset is **MFI**, which is expected due to the industrial relevance of several important materials with that zeolite structure including ZSM-5, silicalite-1, and TS-1.^47,55^ Other common frameworks include industrially important and well-studied zeolites with multiple chemistry types including **CHA**, *BEA, **AFI**, and **FAU**.^56^ Multiple possible chemistries, coined “zeotypes”, give rise to frameworks with different heteroatoms, including AlPOs, SAPOs, germanosilicates, borosilicates, and other metal-containing structures (Ti, Fe, Co, V, Zn, Sn, etc.), as shown in different colors in Figure S1. Clearly, small and medium pore sizes are dominated by aluminosilicates (blue). However, exceptions do exist. The **AEN**, **AEL**, and **AFO** frameworks primarily exist as AlPOs (green), while the **ITH** and **STW** frameworks are purely siliceous (orange) or germanosilicates (red). For example, the SAPO form of **AEL** has been successfully deployed for applications in dewaxing and fuel upgrading,^57,58^ hence potentially biasing the reported synthesis routes in the literature toward AlPO chemistry. From the rightmost two plots in Figure S1, one can clearly observe that large and extra-large pore zeolites have a markedly higher frequency of being synthesized as zeotypes other than aluminosilicates, such as germanosilicates (**BEC**, **IWR**, **IWW**, **UTL**, **IRR**, *CTH), AlPO (**ATO**, **-CLO**, **IFO**) and borosilicates (*-SVY, **SFH**). The increased frequency of germanosilicates can be rationalized by the important role of Ge in stabilizing large pores due to more flexible Ge–O–Ge bonds.^39,59^

The dataset provides insight into the chronological progression of zeolite research as it traces the total number of reported synthesis routes over time. As shown in Figure 1c, initially, research focused on small-pore frameworks (orange), followed by medium- and large-pore frameworks (blue and green). Extra-large-pore frameworks (purple), documented much later in the 1990s, account for a considerably smaller number of synthesis routes. Extra-large-pore frameworks have lower thermodynamic stability compared to their small/medium-pore counterparts, and they require careful design of bulky OSDAs,^60,61^ resulting in complex and expensive organic molecules, thus rendering their synthesis more challenging.

Another important feature of the dataset is the presence of negative data. Many scientific fields often suffer from the underreporting of negative or “failed” data, leading to the literature being skewed toward positive results, which can bias perceptions of chemistry and hinder scientific advancement. The zeolite synthesis scientific literature reports negative results, such as amorphous or dense crystalline phases, alongside successful ones. As such, ZeoSyn includes synthesis conditions resulting in a failed synthesis (dense and/or amorphous phases as the final product), which constitutes approximately 25% of the dataset.

#### Gel Composition

Figure 1d shows the distribution and range of several important gel compositional ratios, including molar ratios between heteroatoms (Si/Al, Si/Ge, Si/B, Si/Ti, and Al/P). Common Si/Al values typically range from about 5 to 40, although a significant number of synthesis routes take place above or below this range. While conventional zeolite synthesis typically occurs with Si/Al > 1, values below 1 exist in the dataset due to the presence of AlPO- and SAPO-type synthesis.^62^ Among the zeotypes, germanosilicates have the smallest range, with Si/Ge ranging from 2 to 15.^63^ In contrast, titanosilicates have the largest range, with Si/Ti generally going above 25, and occasionally above 100, when syntheses are carried out in fluoride media.^47^Figure 1e represents the mineralizing agents (OH^–^/T, F^–^/T), metal cations (Na^+^/T, K^+^/T), and OSDA (OSDA/T). Noticeably, the ratios of these synthesis factors to T (where T = ∑_*i*_*n*_*i*_ where *n*_*i*_ is the amount of the *i*th heteroatom in tetrahedral sites present in synthesis) are typically below 1, but outliers do exist, representing the utilization of an abundance of that element. As expected, mineralizing agents take on ratios <1, with F^–^/T having a more restricted range compared to OH^–^/T. Across the alkaline metals (M^+^ = Li^+^, Na^+^, K^+^), the range of M^+^/T increases from Li^+^ < Na^+^ < K^+^. This could be tentatively explained by the different solubility of the metal silicates formed in the synthesis media.^64^ OSDA/T typically ranges from 0 to 1 even though the OSDA/T ratios for carefully designed OSDAs are typically close to 0.05. The large range of OSDA/T could be rationalized by the fact that OSDAs are generally introduced in their hydroxide form in excess to regulate the pH of the synthesis gel. Moreover, the use of OSDA in excess also helps to overcome the issue of undesired Hoffman degradation of ammonium-based OSDAs. However, the minimal usage of OSDA (substoichiometric levels) could be crucial when expensive/complex molecules are used.^65^

#### Reaction Conditions

Figure S3a/b shows the distributions of crystallization temperatures and times of different zeotypes and different pore sizes. For aluminosilicates (blue in Figure S3a), crystallization temperatures of aluminosilicates are broad and bimodal in nature, indicating that some aluminosilicates can be synthesized at lower temperatures, as observed by the secondary peak at 100 °C. In contrast, AlPOs (red in Figure S3a) tend to be synthesized at much higher temperatures compared to aluminosilicates, with some even exceeding 200 °C. The reason for that is unclear, but it could be because of the different synthesis media required for AlPO-type materials (acidic media) compared to classical aluminosilicates (basic media). Thus, the different nucleation–crystallization mechanisms would require different crystallization temperatures to facilitate the mobility of heteroatoms. Moreover, high temperatures under alkaline conditions would result in Hoffman degradation of OSDAs, thus limiting the use of high temperatures in the synthesis of aluminosilicates. In addition, the opposite trend applies for crystallization time, where the AlPO syntheses typically take a much shorter amount of time compared to aluminosilicates (red vs blue in Figure S3c). Other zeotypes such as germanosilicates (orange) and borosilicates (green) have moderate crystallization temperatures but with a much smaller range of values from 150 to 180 °C. In addition, the crystallization temperature is also correlated with pore size. Figure S3b shows that the median (white dot) temperature increases with pore size from small < medium < large < extra-large pore zeolites. Higher reaction temperatures may be required to synthetically access higher energy states corresponding to larger-pore zeolites (less stable compared to smaller-pore zeolites).

In Figure S4, we examine the relationship between crystallization temperature and framework density for different zeotypes across different pore sizes. We observe a positive relationship between the two variables for the small (orange) and large (green) pore frameworks across different zeotypes. Higher crystallization temperatures allow the synthesis to overcome the energy barrier associated with the formation of these more thermodynamically stable structures with higher framework density.^66^ This phenomenon aligns with Ostwald’s rule of stages, where the systems often pass through metastable states before settling into their most stable form. Consequently, as metastable structures gradually evolve, they transition to more thermodynamically favorable frameworks.^67^ However, this positive correlation should be regarded more as a rule-of-thumb, offering experimentalists a starting point for selecting higher temperatures when seeking to crystallize materials with a higher framework density. The positive correlation between crystallization temperature and framework density is not always observed, especially when there is no clear trend in medium (blue) and extra-large (purple) pore zeolites, as zeolite crystallization is governed by the complex interplay between reaction temperature and other factors, such as gel composition and OSDA.

#### Organic Structure-Directing Agents (OSDAs)

**Figure 2:**
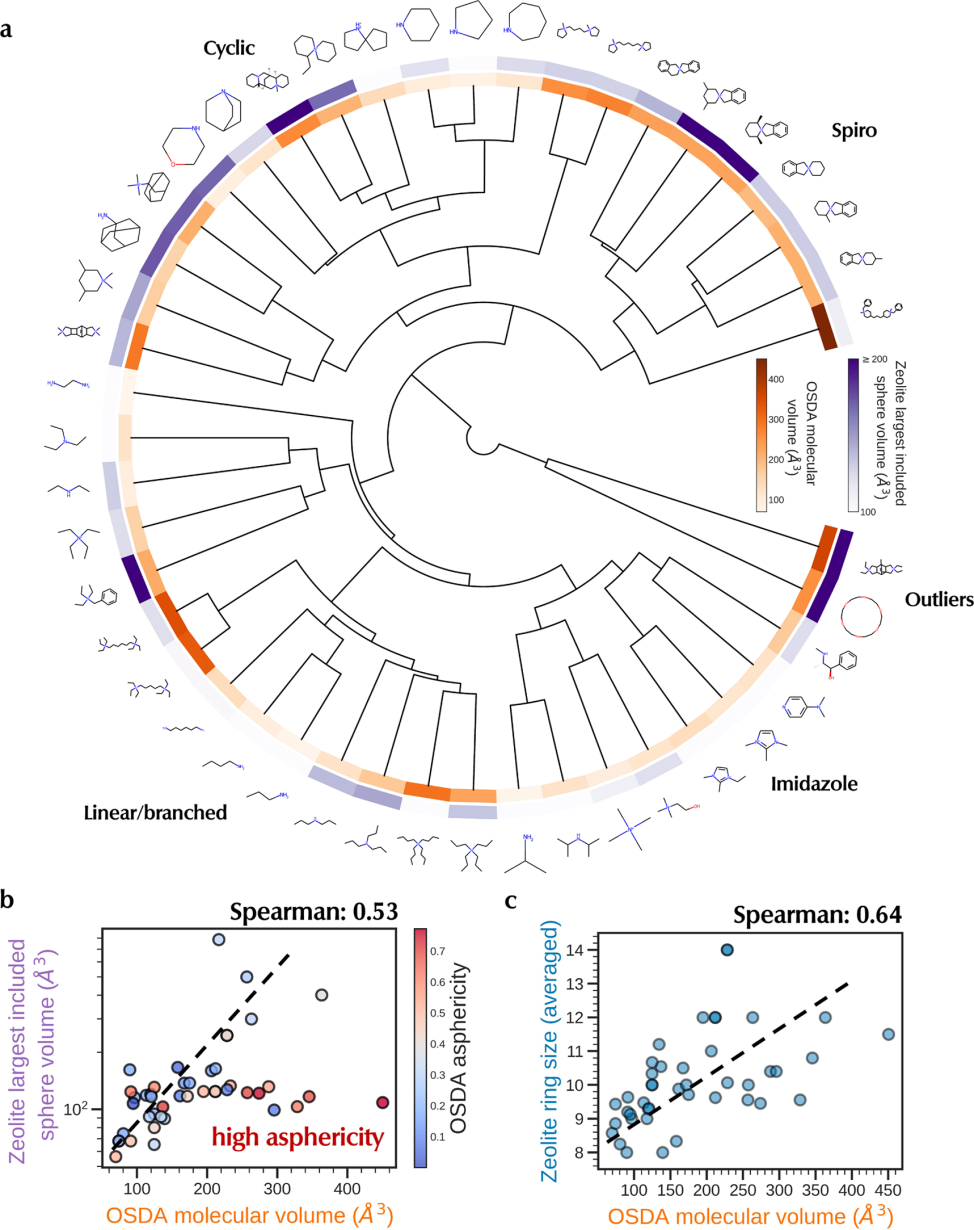
OSDAs in ZeoSyn dataset. (a) Hierarchical clustering of the top 50 most frequent OSDAs in the dataset, labeled with the main classes of molecular structures. Splits are obtained through agglomerative hierarchical clustering of OSDA Morgan fingerprints.^68^ Each OSDA is colored by its molecular volume (orange), and the median largest included sphere of zeolites formed by the OSDA (purple). The concomitant intensities of the colors show a positive correlation between the two properties. (b) Positive correlation between zeolite largest included sphere vs OSDA volume. Red points refer to high asphericity, which accounts for outliers. (c) Positive correlation between zeolite ring size vs OSDA volume.

OSDAs play an indispensable role in zeolite synthesis, as they act as templates, guiding the arrangement of building blocks to form a porous zeolite framework. Shape, size, flexibility, hydrophilicity, and charge distribution of the OSDA, among other factors, strongly influence zeolite crystallization kinetics and hence phase specificity.^10,11,69^Figure 2a shows the most frequent OSDAs present in the dataset, organized in a dendrogram obtained from hierarchical clustering of OSDA Morgan fingerprints.^68^ The clustering analysis reveals that the predominant classes of OSDAs are ammonium cations, characterized by linear-chain and cyclic groups. Imidazole derivatives and spiro-type ammonium are frequently used in zeolite synthesis due to their rigid structure, ease of synthesis, and cheap precursors.

With both OSDA and zeolite products present in the synthesis route, the ZeoSyn dataset allows for insights into the zeolite–OSDA relationships. One key example is visualized in Figure 2a, where the orange ring shows the OSDA molecular volume (in 3D) and the purple ring shows the zeolite largest included sphere in frameworks formed by using the OSDA, which is an approximation of the zeolite pore size (some pores are not spherical). The concomitant intensities of both properties show that OSDA volumes (dark orange) are positively correlated with the pore volume of zeolite product (dark purple). At face value, this can be rationalized by bulkier OSDAs being used to template frameworks with larger pore sizes.^10,11,70^ For example, the spiro amines (top right) have high molecular volumes due to the spiro scaffold, which, in turn, tends to give zeolite frameworks with large pores or cavities.

In Figure 2b, we observe that the Spearman rank coefficient, which measures the statistical dependence between the rankings of two variables,^71^ is positive at 0.53, thereby confirming a positive correlation between OSDA molecular volume and zeolite pore volume. This trend is particularly evident for OSDAs with low to medium asphericity (gray/blue points). However, there are exceptions to this trend. Outliers can be explained by recognizing that the volume of OSDA alone does not fully account for its templating effect. Other factors like shape, flexibility, and charge are also crucial in determining the OSDA’s ability to template specific zeolite frameworks. For instance, outliers of the positive trend can be explained by their high asphericity (i.e., red points in Figure 2b), meaning that they are highly nonspherical and asymmetric in shape. For these OSDAs, the assumption of the largest sphere breaks down, resulting in a deviation from the positive trend. This highlights the potential pitfall of designing OSDAs based only on the largest included sphere of a zeolite pore and hence underpins the importance of considering the pore shape.

In the same vein, we visualize the relationship between zeolite ring size vs OSDA volume in Figure 2c, which also reveals a positive correlation (Spearman coefficient of 0.64). Again, this aligns well with domain knowledge, where larger ring sizes tend to require larger OSDAs. Outliers may be rationalized by the fact that smaller OSDAs such as tetramethylammonium may play a space-filling role instead of acting as a true template. Regardless, despite the complexity of zeolite–OSDA relationships, the ZeoSyn dataset allows for such visualizations that could inform the design of OSDAs with optimal molecular volumes for synthesizing hypothetical zeolites with specific cavity/ring sizes.

#### Competing Phases and Intergrowths

Since some zeolites are metastable with respect to each other, a single synthesis may result in two or more crystalline phases being formed, possibly through different simultaneous kinetic processes.^72^ Fortunately, the zeolite literature often reports secondary phases formed. As such, the ZeoSyn dataset also captures competing zeolite phases, where it reports the presence of reaction side products or zeolite intergrowths. For example, frequently observed pairs of competing phases are **TON** & **MFI**, **FAU** & **GIS**, and **AFI** & **SOD**, as shown in Figure S5. This creates an opportunity to model competing phases for zeolites. Zeolite intergrowths consist of two phases interacting through stacking faults in one or more directions, resulting in an alternation between the frameworks. This is a result of both phases having matching lattices, which allows for nucleation of both phases in the absence of grain boundaries.^73^ In the ZeoSyn dataset, common intergrowths include **ISV**/**BEC**, **ERI**/**OFF**, and **MFI**/**MEL**, as shown in Table S2. We will discuss how machine-learning rationalization of ZeoSyn can help achieve phase-selective synthesis between competing phases and inform the synthesis of intergrowths through a zeolite framework prediction model.

### Zeolite Framework Prediction Model

Zeolite synthesis is a high-dimensional space (gel composition, organic templates, and reaction conditions) with complex structure–synthesis relationships. For instance, the large number of degrees of freedom of a flexible OSDA makes it difficult to selectively template a specific zeolite framework,^10,11^ underscoring the need for a comprehensive evaluation of zeolite synthesis. There is potential for ML models to learn from these high-dimensional data to capture quantitatively synthesis trends beyond what is currently understood by domain experts.

**Figure 3:**
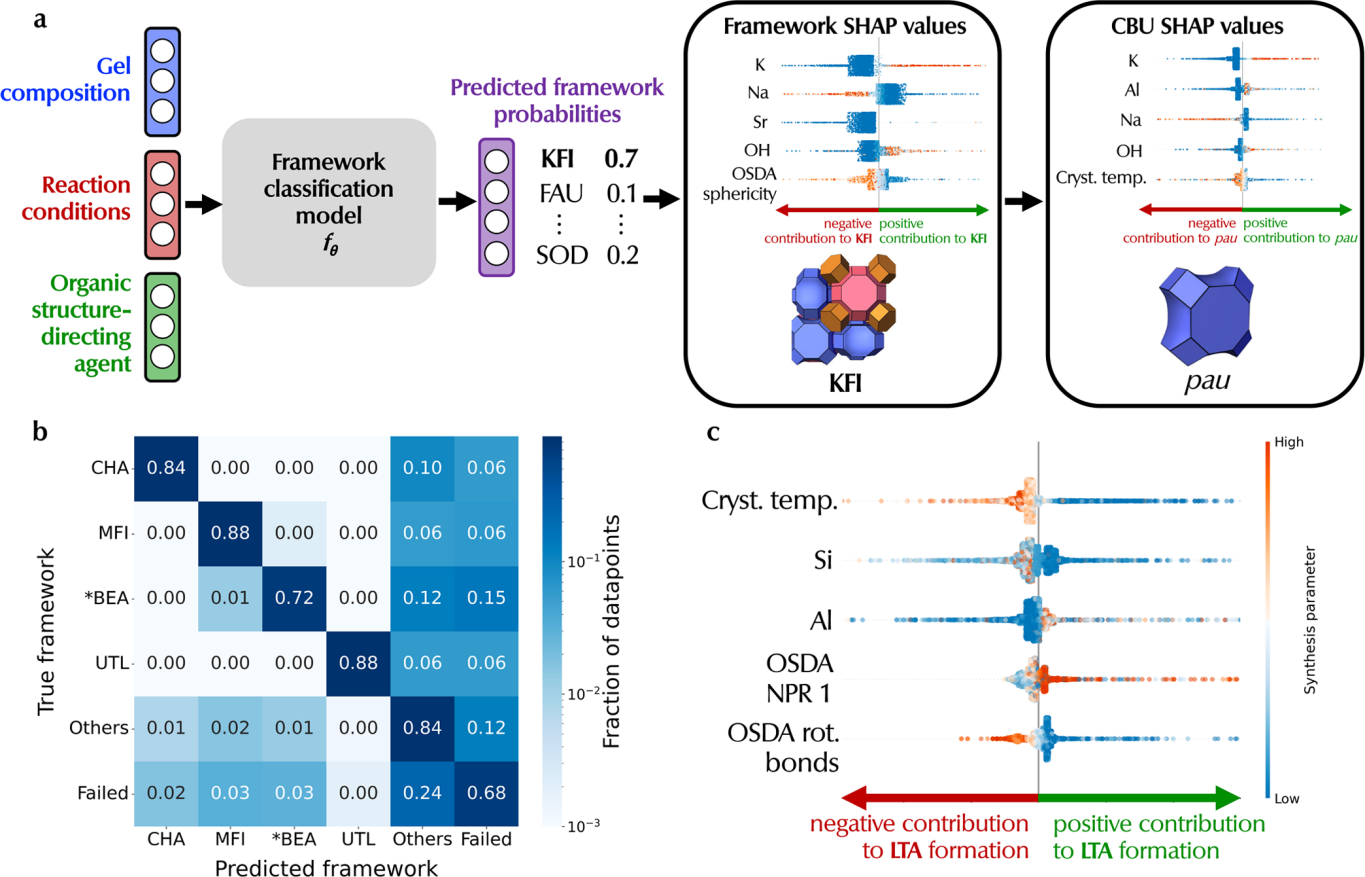
Interpretable ML framework for synthesis–structure relationships. (a) Schematic of zeolite phase predictor model. Given the synthesis parameters, the model *f*_θ_ predicts the resultant framework (e.g., **KFI**). Additionally, if a dense or amorphous phase is expected, the model predicts a “Failed” class. The predicted framework probabilities are used to calculate framework-level SHAP values. In addition, CBU-level SHAP values of composite building units (CBUs) are obtained by aggregating framework-level SHAP values, allowing for CBU-level analysis of synthesis parameters. (b) Normalized confusion matrix of the phase predictor model. Here, we have selected one representative small (**CHA**), medium (**MFI**), large (*BEA), and extra-large pore (**UTL**) framework. “Others” refers to all other frameworks, while “Failed” refers to amorphous/dense phases. (c) An example of a framework-level SHAP analysis quantifying the positive/negative impact of synthesis parameters on the probability of **LTA** framework formation.

We develop a ML classification model *f*_θ_ to predict the zeolite product (e.g., **LTA**) given synthesis parameters (gel composition, reaction conditions, and OSDA) as shown in Figure 3a. We also leverage negative data by including failed synthesis (dense/amorphous phases) in the training data for the model to learn regions in synthesis space where a specific zeolite framework has a higher probability to crystallize.

#### Model Implementation

We select the random forest architecture as it is computationally efficient and offers strong performance on tabular datasets compared to deep learning architectures.^74^ We train a random forest model on the ZeoSyn dataset with the inputs shown in Figure 3a. The gel composition is represented by the relative molar fractions of elements (e.g., Si) present in the gel. For reaction conditions, only the crystallization time and temperature are used. For OSDA, although some syntheses employ two or more OSDAs, we consider only OSDA with the largest molecular volume. We featurize the OSDAs using their physicochemical descriptors (e.g., molecular volume and 2D shape descriptors).^31^ The full list of OSDA features and their descriptions can be found in Table S1.

#### Model Performance

The model is evaluated on held-out, unseen test syntheses (from random split) on the framework prediction task with a model accuracy of 0.73. We note that previous work to predict the zeolite framework given synthesis parameters as reported by Muraoka et al., albeit on a much smaller scale,^29^ reported an overall accuracy of 0.82. Our reported accuracy of 0.73 is lower, which can be rationalized by the significant difference in the number of zeolites between ZeoSyn and the dataset used by Muraoka et al. Specifically, in Muraoka et al., the number of zeolite classes to predict is only 23. In contrast, we predict 1 out of 220 possible classes (an order of magnitude larger). In addition, the work by Muraoka et al. focused on OSDA-free synthesis versus the work presented here, which includes OSDA-mediated synthesis routes.

The confusion matrix shown in Figure 3b highlights classification performance on one representative small (**CHA**), medium (**MFI**), large (*BEA), and extra-large (**UTL**) pore framework, with “Others” referring to all other frameworks aggregated together (for ease of visualization) and “Failed” referring to dense/amorphous phases. Most predictions fall on the diagonal of the confusion matrix, indicating a high prediction accuracy of 0.68–0.88 for these classes. Notably, the model does the best on the **MFI** framework, possibly due to its relatively high number of synthesis routes as previously shown in Figure S1. Even for the less common, extra-large-pore **UTL** framework, the classification accuracy is still high, at 0.88, showing that the model can generalize to frameworks of different pore sizes. As shown by the high-intensity off-diagonal elements being on the right-hand side of the matrix, the majority of the errors made by the model are misclassifications as another framework (“Others”) or dense/amorphous dense (“Failed”). This is expected as the number of data points in “Others” is large due to the aforementioned aggregation (consisting of 215 frameworks). The same is true for the “Failed” class, where there are over 4000 data points. Moreover, the model also shows strong performance in discriminating different pore sizes as shown in Figure S6 with high accuracies of 0.78–0.86. This shows that the model can accurately predict the resultant framework product, given a set of synthesis parameters.

### Machine-Learning Rationalization of Zeolite Synthesis Parameters

Beyond providing accurate predictions of the reaction product, we analyze the synthesis knowledge learned by the classification model to rationalize the impact of synthesis parameters on the formation of a specific zeolite framework. As such we implement SHapley Additive exPlanations (SHAP), a game-theoretic approach to explain the output of ML models through optimal credit allocation with local explanations,^75^ on the classification model. For each prediction, we calculate SHAP values to determine the impact of each synthesis parameter on the probability of forming a specific zeolite framework. A synthesis parameter with positive SHAP values increases the probability of the formation of a zeolite framework. For instance, the first row of Figure 3c uncovers a physically grounded trend that low crystallization temperatures (blue points) have positive SHAP values (increases probability of **LTA** formation). Conversely, high temperatures (orange points) have negative SHAP values (decreases the probability of **LTA** formation). This would agree with the fact that **LTA** is a small-pore zeolite with a relatively low framework density hence requiring low crystallization temperatures (at least for OSDA-free synthesis of low-silica **LTA**) as previously discussed in Figure S4. Importantly, these SHAP values allow researchers to peek into the high-dimensional synthesis knowledge learned by the model, which can provide valuable insights into the zeolite crystallization process by providing SHAP values associated with any synthesis parameter in a *low dimension* that are readily interpretable by a human expert. In this section, we quantify the impact of synthesis parameters at two different levels of zeolite structure:

1. **Framework-level SHAP** shows the positive/negative impact of a synthesis parameter on the probability of crystallizing a specific zeolite framework (e.g., **KFI** in Figure 3a)
2. **CBU-level SHAP** shows the positive/negative impact of a synthesis parameter on the probability of forming a structure that contains a specific composite building unit (CBU) (e.g., *pau* cage in Figure 3a)

#### Framework-Level SHAP

**Figure 4:**
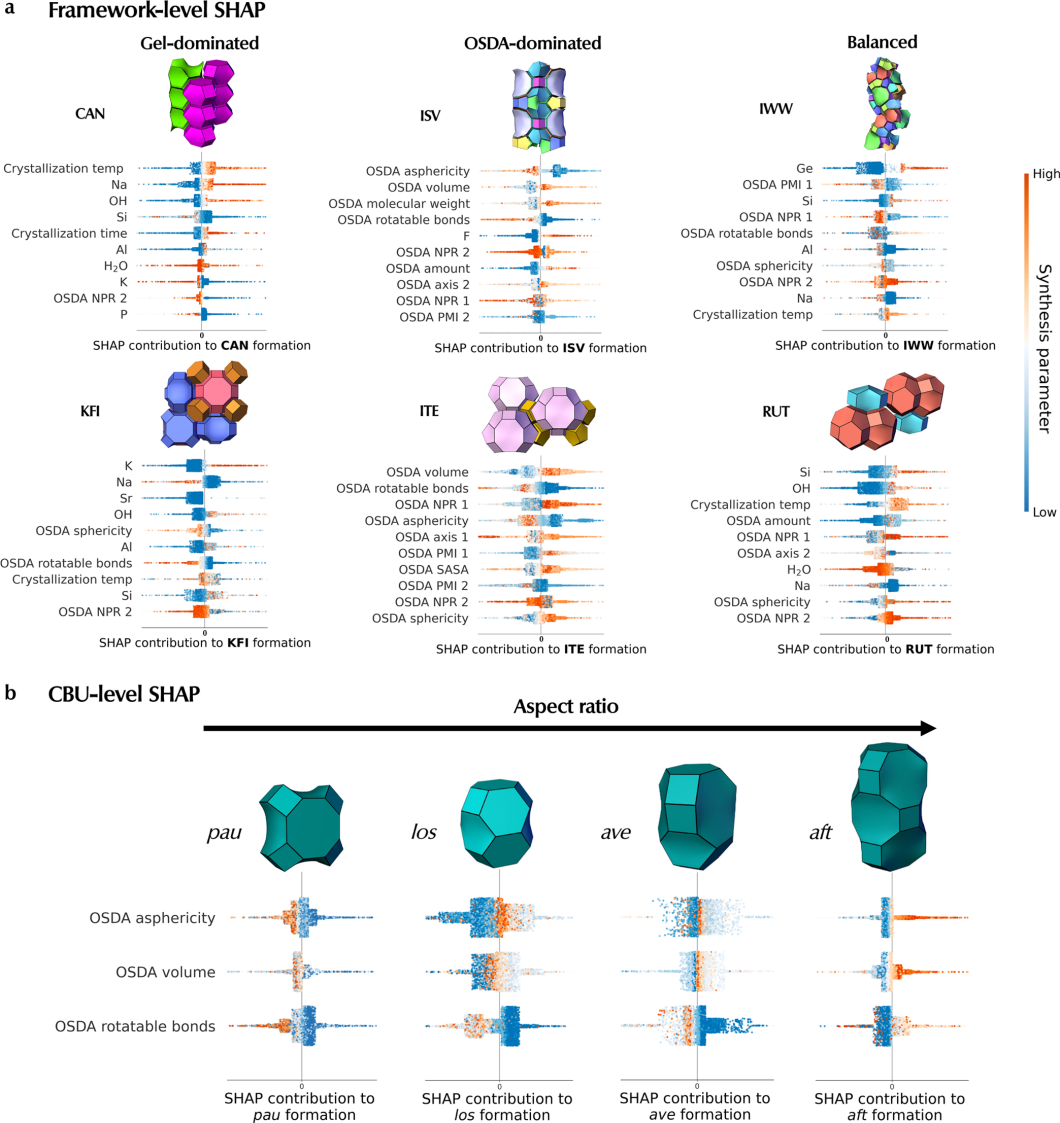
Revealing key synthesis–structure relationships. (a) Framework-level SHAP analysis revealing the top 10 (out of 43) most important synthesis parameters favoring the formation of specific frameworks. Each framework belongs to 1 out of 3 types of synthesis based on its top synthesis parameters: 1) gel-dominated synthesis (**CAN**, **KFI**) where most top parameters are inorganic-related, 2) OSDA-dominated synthesis (**ISV**, **ITE**) where most top parameters are OSDA-related, and 3) balanced synthesis (**IWW**, **RUT**) where even attribution is given to inorganic and OSDA parameters. Every point is an individual synthesis colored by the value of the synthesis parameter (orange and blue colors indicate high and low values, respectively). (b) CBU-level SHAP analysis of large CBUs showed OSDA parameters favoring their formation.

Framework-level SHAP identifies the most important synthesis parameters driving the formation of a specific zeolite framework. Larger positive/negative SHAP values correspond to larger positive/negative changes in the probability of obtaining a specific framework given the synthesis parameter. Here, we consider all 43 inputs of the model *f*_θ_ and show only the top 10 most important synthesis parameters (in descending order) for specific frameworks as shown in Figure 4a. This ordering of synthesis parameters is determined by the mean absolute value of the SHAP values corresponding to the parameter.

We note the two different types of synthesis parameters: 1) inorganic, which relate to composition of the inorganic components of the synthesis gel (e.g., Si, Al, OH^–^, F^–^, etc.), and 2) OSDA, which relate to the organic template (e.g., OSDA volume, OSDA rotatable bonds, etc.), as shown in Table S1. Consequently, this allows us to categorize the formation of a specific zeolite framework as 1 out of 3 main types of synthesis based on its top synthesis parameters as shown in Figure 4a: 1) gel-dominated synthesis where most top parameters relate to inorganic components, 2) OSDA-dominated synthesis where most parameters relate to the OSDA, and 3) “balanced” synthesis where even attribution is given to both inorganic and OSDA parameters. An exhaustive list of framework-level SHAP for all known frameworks has been included in Figure S12–S18.

##### Frameworks with Gel-Dominated Synthesis

These frameworks have syntheses where inorganic components play a more crucial role, with few (≤3 out of the top 10) OSDA-related parameters. Figure 4a shows two such frameworks (**CAN**, **KFI**). In terms of the gel composition, **CAN** and **KFI** share the common trend that both are favored by high levels of the mineralizing agent OH^–^. However, beyond that, many gel components have vastly different impacts on these two frameworks. For instance, such analysis reveals **CAN** formation seems to be favored by high Na^+^ and low K^+^.^76^ Conversely, **KFI** formation follows the opposite trend, where it appears to be favored by low Na^+^ and high K^+^.^77^ In terms of reaction conditions, high and low crystallization temperatures favor **CAN** (due to high framework density) and **KFI**, respectively.^78^

##### Frameworks with OSDA-Dominated Synthesis

These frameworks have syntheses where OSDA features are more important. As shown in Figure 4a, both **ISV** and **ITE** have all of their top synthesis parameters related to the OSDA. One notable exception is the high amount of F^–^ driving **ISV** formation due to the presence of the *d4r* CBU in the framework.^79^ We observe that OSDAs favoring these two frameworks have low asphericity (indicating the need for a spherical OSDA), high volume, and a small number of rotatable bonds (indicating rigidity). However, differences do exist: **ITE** formation is associated with high values of OSDA NPR 1 (first normalized principal moment of inertia ratio) with the orange points clearly on the right-hand side, while this effect is not present in **ISV** formation where orange and blue points overlap one another. Moreover, unlike **ITE**, **ISV** requires higher amounts of OSDA. We hypothesize that such insights into the influence of OSDA on the synthesis outcome could be used to guide the design of optimal OSDAs that target a specific framework.

##### Frameworks with Balanced Synthesis

These frameworks have been synthesized by a balance of inorganic and OSDA components. It is evident from the gel composition parameters (Figure 4a) that high Ge promotes **IWW** formation, which can be rationalized by Ge’s role in stabilizing the *d4r* cage.^80^ In contrast, **RUT** requires a high Si content, which could be expected considering its dense structure. In addition, high Na^+^ disfavors both frameworks, albeit the impact of the Na parameter is ranked much lower. Inspection of the OSDA sphericity in both frameworks reveals an opposing trend: **IWW** and **RUT** are favored by low and high OSDA sphericity, respectively. This could be explained by the large spherical cavity present in **RUT** (see Figure S7a), while **IWW** mainly consists of long channels (see Figure S7b) that require longer, less spherical OSDAs.

#### CBU-Level SHAP

We also consider the synthesis parameters that contribute to the formation of the specific building units that make up the frameworks. Zeolites adopt a hierarchical structure, where CBUs combine to form the zeolite frameworks themselves. SHAP analysis using a CBU-centric treatment may enable the analysis to extrapolate to hypothetical (beyond the scope of this work) frameworks. To obtain CBU-level SHAP values of a specific CBU, we employ an aggregation approach of summing the SHAP matrices of all known zeolite frameworks that contain that CBU as described in the Methods section. This amplifies SHAP values of important factors contributing to common building units while suppressing those that are less important, giving rise to a CBU-centric view of the synthesis parameters. An exhaustive list of CBU-level SHAP for all CBUs reported on IZA has been included in Figure S10 (small CBUs) and Figure S11 (large CBUs).

##### Small CBUs

We uncover the most important inorganic parameters driving the formation of a selection of 4 small CBUs in Figure S8. As shown, the synthesis of small CBUs is all inorganic/gel-dominated instead of OSDA-dominated with the top 5 parameters relating to the inorganic components. We observe the well-established fact that high Ge and F^–^ are ranked as the top parameters contributing to *d4r* formation. Furthermore, this analysis reveals a less obvious relationship where a low crystallization temperature also positively influences *d4r* formation. Similarly, *d8r* is favored by low crystallization temperatures, but is mainly promoted by high K^+^ and Cs^+^ cations.^81^*can* is driven by high K^+^ and requires large amounts of OH^–^ as a mineralizing agent. Lastly, high Na^+^ and low crystallization temperatures favor *gme* formation.^82^

##### Large CBUs

In contrast to small CBUs, the formation of large CBUs is influenced by OSDA parameters due to the need for a structure-directing effect by OSDAs. Figure 4b shows a series of large CBUs (≥30 T sites) with an increasing aspect ratio (*pau* < *los* < *ave* < *aft*). Interestingly, in the first row, CBU-level SHAP discovers a clear relationship between the aspect ratio of the CBU and OSDA asphericity (a measure of the deviation from sphere). For *pau*, low OSDA asphericity (dark blue on positive side) gives rise to positive SHAP values, indicating the need for a spherical OSDA. Indeed, this is due to the symmetrical shape of the *pau* cage. Consequently, when one considers cages with medium-level aspect ratios (*los*, *ave*), one can observe neither very high (orange) nor very low levels (blue) of OSDA asphericity promote their formation. Instead, it is medium levels (light blue on the positive side) of OSDA asphericity that drive their formation. As we transition to a CBU with an even higher aspect ratio (*aft*), now only high levels of OSDA asphericity (orange) are needed to drive its formation, indicating the increasing need for longer, more asymmetric molecules to template CBUs with an increasing aspect ratio. Similarly, the same trend also applies for OSDA volume (second row) as the aspect ratio of the CBU increases, suggesting that larger/bulkier OSDAs would facilitate the formation of cavities with larger aspect ratios. Lastly, in the last row, SHAP reveals a rather surprising trend: The first three CBUs (*lta*, *los*, *ave*) are favored by a very low number of OSDA rotatable bonds, which suggests the need for rigid molecules. Surprisingly, the opposite trend exists for *aft*, where there is a need for a more flexible OSDA with high aspect ratio (e.g., hexamethonium).^83^

#### Applications of SHAP Analysis

We suggest the utility of the aforementioned SHAP analysis on two important applications in zeolite synthesis: 1) competing phases, where the goal would be to obtain a single, pure framework, and 2) intergrowths, where the goal would be to obtain a product with 2 zeolite phases intergrown into each other. Here, we apply framework-level SHAP to inform on rational design for the above 2 goals.

##### Competing Phases

**Figure 5:**
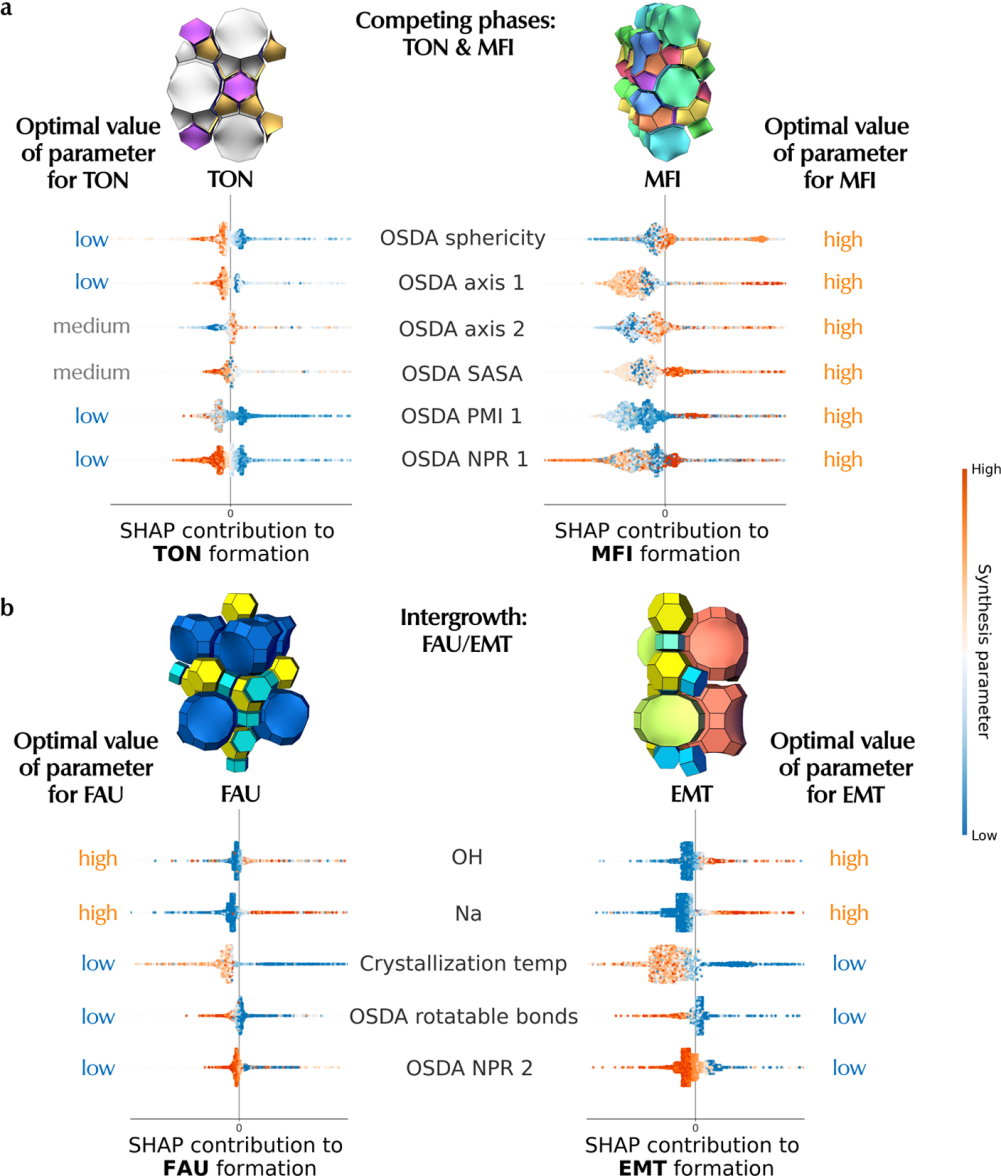
Application of interpretable ML framework on (a) competing phases (**TON** and **MFI**). The left- and right-most columns describe the optimal value of the OSDA parameter for maximizing the formation probability of **TON** and **MFI**, respectively. For example, the first row shows *opposing* effects of the OSDA sphericity: low OSDA sphericity promotes **TON** formation while suppressing **MFI** (and vice versa). (b) Intergrowth (**FAU**/**EMT**), where the task may be to maximize the formation of both frameworks. For example, the first row shows *aligne* effects of OH^–^: high OH^–^ promotes both **FAU** and **EMT**, which may lead to **FAU**/**EMT** intergrowth. This shows framework-level SHAP could be a powerful way to inform domain experts on the rational design of synthesis parameters to control phase selectivity.

We consider the most common pair of competing phases in the ZeoSyn dataset, **TON** & **MFI** (Figure S5), where these 2 frameworks are frequently formed in the same synthesis. **MFI** is a framework that often appears as a competing phase due to its ease of synthesis and wide synthesis window. Here, we consider achieving the phase-selective synthesis of **TON** in the absence of **MFI**. Figure 5a shows the framework-level SHAP for the **TON** and **MFI** frameworks. To achieve a phase-selective synthesis of **TON**, we inspect the impact of OSDA sphericity (first row) on the two frameworks, which reveals *opposing* effects on the frameworks: clearly, an OSDA with low sphericity promotes **TON** formation while suppressing **MFI** as indicated by the rightmost column. In the same vein, the other factors relating to OSDA, such as axis 1, axis 2, solvent-accessible surface area (SASA), principal moment of inertia (PMI 1), and first normalized principal moment of inertia ratio (NPR 1) all show *opposing* effects for the 2 frameworks. Beyond OSDA-related parameters, this analysis can also be extended to identify important inorganic parameters for another common pair of competing phases (*BEA and **BEC**). Figure S9 shows that Si/Ge, crystallization temperature, and time are the key parameters to achieve phase-selective synthesis. As such, this showcases framework-level SHAP as a powerful tool for identifying promising synthesis “knobs” and recommends the appropriate direction to tune these “knobs” for phase-selective synthesis.

##### Intergrowths

It is highly desirable to synthesize intergrowths, as they combine the advantages associated with two different frameworks. Here, the goal is to promote the crystallization of 2 frameworks within the same crystal. As such, we can flip the switch and identify “knobs” that have *aligned* (instead of opposing in the case of competing phases) effects on the formation of the 2 constituent frameworks. For example, in Figure 5b we consider a known zeolite intergrowth **FAU**/**EMT**, which shows the common parameters such as high OH^–^, high Na^+^, low crystallization temperature, low number of OSDA rotatable bonds (rigid OSDA) and low OSDA NPR 2 as potential synthesis parameters to tune to favor the formation of a **FAU**/**EMT** intergrowth. Indeed, such analysis is corroborated by the following 3 aspects of a reported synthesis of a **FAU**/**EMT** intergrowth.^84^ First, 18-crown-6 is used as the OSDA, which has no rotatable bonds due to its cyclic structure. Second, the synthesis employed relatively high levels of Na^+^ (Na^+^/T = 0.31–0.46). Third, a relatively low crystallization temperature of 100 °C was used. These 3 observations in the reported synthesis are well aligned with recommendations suggested by framework-level SHAP in Figure 5b. As such, this is a testament to the usefulness of such analysis as a tool for not only understanding key parameters impacting the crystallization of intergrowths but also a step toward the rational design of their synthesis.

We are cognizant that SHAP quantifies only the impact of synthesis parameters on a local level. At times, strong correlations in the dataset may lead to erroneous conclusions in the SHAP analysis. For instance, if two CBUs frequently co-occur in multiple frameworks, it may be challenging for the CBU-level SHAP to accurately assign the impact of synthesis parameters toward one of them. Moreover, this approach only hints at which synthesis parameter(s) and the direction one may modify it to achieve a certain target in zeolite synthesis. Since SHAP gives local explanations that relate to one synthesis parameter at a time, it does not suggest the exact values for multiple synthesis parameters jointly given a desired zeolite. Given the scope of this work is confined largely to the rationalization of synthesis parameters using the ZeoSyn dataset, future work will focus on the design of synthesis routes, where one could formulate this as a synthesis route prediction task. This approach could be particularly valuable for zeolites with narrow synthetic intervals.

## Conclusion

In this work, we extract over 50 years of published zeolite synthesis data, including gel composition, reaction conditions, precursors, OSDAs, and zeolite structure, from the zeolite literature, giving rise to ZeoSyn: the largest comprehensive zeolite synthesis dataset reported in the literature to date. Visualizations of synthesis parameters explain the physical trends in zeolite synthesis while uncovering notable exceptions. We also showcase the utility of the ZeoSyn dataset by training a framework classification model that has shown strong performance in predictive accuracy. The main utility of the model lies in the subsequent SHAP analysis, which has been shown to be a powerful approach to uncovering the impact of the most important synthesis parameters for a specific framework or composite building unit. Importantly, it is worth noting such insights are enabled by the unprecedented scale of the dataset. Furthermore, this approach has been shown to be useful for informing the design of synthesis parameters for phase-selective and intergrowth synthesis. It is hoped that the scale and coverage of ZeoSyn dataset will enable future efforts in ML modeling of zeolite synthesis and pave the way for data-driven discovery of zeolitic materials.

## Methods

### Data Extraction and Validation

Data extraction techniques used in this paper were built upon previously published work.^28,32,33,35,85^ Briefly, the Elsevier Scopus platform is used to find zeolite articles containing the search terms “zeolite”, “OSDA”, “aluminophosphate”, and “molecular sieve”, resulting in a dataset of approximately 130,000 papers. From this corpus, gel composition, reaction conditions, precursors, OSDA names, reaction products, and reaction product properties are extracted from a paper’s tables and synthesis sections using a combination of table parsing, named entity recognition modeling, regular expressions, and domain-specific keyword matching. Each extracted synthesis route is manually checked to ensure accuracy and to remove false positives. OSDAs and zeolite structures are featurized in the same fashion as our previous work,^32^ where OSDA names are standardized to a canonical SMILES string and featurized with RDKit^86^ and zeolite structures are featurized with structural parameters obtained from the International Zeolite Association (IZA) database.^7^ Manual verification is performed on the dataset as follows: every DOI is reviewed to confirm accurate extraction, and we check the ZeoSyn dataset against the values reported in the “materials”, “experimental”, “synthesis conditions”, and “supporting information” sections. This process is conducted three times to ensure the precision and accuracy of the extracted information.

### Hierarchical Clustering of OSDAs

Hierarchical clustering is an algorithm that clusters data points by merging/splitting them successively, resulting in a dendrogram/tree representing the hierarchy of clusters. The root of the tree is the cluster that gathers all of the samples, while the leaves are the clusters with only a single sample. In the context of agglomerative hierarchical clustering, each data point starts as its own individual cluster. The algorithm begins with a forest of clusters that have not been used in the hierarchy being formed. At each iteration, the two closest clusters (according to a distance metric) are combined to form a larger cluster.

A distance matrix *d* is maintained at each iteration. The *d*_*i*,*j*_ entry refers to the distance between cluster *i* and *j* in the initial forest. There are |*u*| and |*v*| observations in clusters *u* and *v*, respectively. Here, we calculate the Euclidean distance between clusters *u* and *v* using the averaging method:^87^
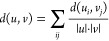
1When two clusters *s* and *t* are combined into a common cluster *u*, they are removed from this forest, and the new cluster *u* is added to this forest. This process repeats until all data points form a single common cluster. Since the goal is to cluster OSDAs according to their molecular structure, each OSDA is featurized by its Morgan fingerprint^68^ using the rdkit.Chem package. Subsequently, hierarchical clustering of OSDAs is implemented using the cluster.linkage function from scipy package^88^ to construct the dendrogram.

### Zeolite and OSDA Featurization

The zeolite structural properties (e.g., ring sizes, largest included sphere) are obtained from the IZA database.^7^ Zeolite frameworks and CBUs are visualized using the 3dt and ToposPro software, with some CBUs graphics obtained from the IZA database.^7^ We featurize the OSDA using its physicochemical descriptors (e.g., molecular volume and 2D shape descriptors) of the organic molecule.^31,32^ The full list of OSDA features and their descriptions can be found in Table S1. Periodic table in Figure 1c is generated using code from Huo et al.^89^

### Zeolite Framework Prediction Model

We train a supervised classification model using random forest to predict a zeolite framework product given a synthesis recipe. This choice of modeling is motivated accordingly: 1) tree-boosting models offer competitive performance on tabular datasets like ZeoSyn; 2) the scale and coverage of the ZeoSyn dataset (not the model architecture) enables good classification performance; and 3) using a tree-based model allows fast computation of SHAP values (by reducing the complexity of exact Shapley value computation from exponential to polynomial time^90^). The model takes in a 43-dimensional vector as input where each element corresponds to either gel composition (e.g., Si, Al, P, etc.), reaction condition (e.g., crystallization time), or an OSDA descriptor (e.g., molecular volume). The model predicts (1 out of 220 classes) a zeolite framework. An 80/20 random train/test split is employed. Since the focus is on the subsequent SHAP analysis, we trained the model with default parameters. It is worth noting that the total number of classes (220 frameworks) is fewer than the number of synthesized frameworks (264) as some frameworks may be reported in patents (outside the scope of this work) but not in the scientific literature.

### SHAP Analysis of Zeolite Formation

To analyze the outcomes of the classification model (depicted in Figure 3a), we employ SHAP,^75^ which is a generalized measure for the impact of features. This approach uses Shapley values from game theory to compute the contribution made by each feature to the model prediction. Features are likened to participants in a “game” representing the prediction task, and the SHAP values measure how much prediction is attributed to these features. These values signify the relative importance of a specific feature and its impact on classification. For example, as shown in Figure 3c, SHAP values reveal how altering the value of a feature, either increasing or decreasing, affects the model output. This strategy facilitates both localized understanding of individual model explanations and a comprehensive interpretation of the model behavior. In this work, we calculate SHAP values at two levels: **1) Framework-level SHAP** quantifies the impact of synthesis parameters on the formation of a zeolite framework. They are calculated based on the predicted probabilities using TreeExplainer function from the shap package.^90^**2) CBU-level SHAP** quantifies the impact of synthesis parameters on the formation of a composite building unit (CBU). We employ an aggregation approach to obtain CBU-level SHAP values as follows:

#### Aggregated SHAP

Let  be the framework-level SHAP matrix of framework *f* with *n* observations and *m* features. The CBU-level SHAP matrix *S*_CBU_ is given by aggregating framework-level SHAP matrices:
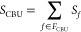
2where *F*_CBU_ is the set of synthesized frameworks containing a specific CBU according to the IZA database.^7^ For example, to obtain CBU-level SHAP matrix *S*_*sod*_ corresponding to the *sod* CBU, we determine the set of frameworks containing *sod*, *F*_*sod*_ = {**FAU**, **SOD**, **LTA**} (note: for the sake of brevity, only 3 *sod*-containing frameworks are listed as more exist). Subsequently, the CBU-level SHAP is given by *S*_*sod*_ = *S*_FAU_ + *S*_SOD_ + *S*_LTA_. Intuitively, by summing up *S*_*f*_ corresponding to frameworks containing the CBU, this aggregation approach amplifies SHAP values corresponding to *common* features that highly impact (positively or negatively) CBU formation while suppressing SHAP values corresponding to the features that do not have much impact. As such, this effectively shifts the SHAP analysis from a framework-centric to a CBU-centric view, allowing for an understanding of factors driving the building units that make up zeolites.

## Data Availability

The corresponding code, dataset, and demo are available online at https://github.com/eltonpan/zeosyn_dataset.

## Supporting Information Available

Information on additional statistics of the ZeoSyn dataset, OSDA physicochemical descriptors, and SHAP analysis on zeolite frameworks and CBUs (PDF)
