# Supplementary material for: ZeoSyn: A Comprehensive Zeolite Synthesis Dataset Enabling Machine-Learning Rationalization of Hydrothermal Parameters
Source: ACS Cent Sci. 2024 Mar 6;10(3):729–43. doi: 10.1021/acscentsci.3c01615 (PMC10979502; doi:10.1021/acscentsci.3c01615)
Supplement: Supplementary file 1 — oc3c01615_si_001.pdf [file oc3c01615_si_001.pdf]

Supporting Information for:

ZeoSyn: A Comprehensive Zeolite Synthesis Dataset Enabling Machine-learning Rationalization of Hydrothermal Parameters

Elton Pan,[†] Soonhyoung Kwon,[‡] Zach Jensen,[†] Mingrou Xie,[‡] Rafael Gómez-Bombarelli,[†] Manuel Moliner,[¶] Yuriy Román-Leshkov,[‡] and Elsa Olivetti[*,†]

[†]*Department of Materials Science and Engineering, Massachusetts Institute of Technology, Cambridge, Massachusetts 02139, United States*

[‡]*Department of Chemical Engineering, Massachusetts Institute of Technology, Cambridge, Massachusetts 02139, United States*

[¶]*Instituto de Tecnología Química, Universitat Politècnica de València-Consejo Superior de Investigaciones Científicas 46022, Valencia, Spain*

Email: elsao@mit.edu

Supporting Information Figures S1–S18

Supporting Information Tables S1–S2

19 pages

Table of Contents

Figures

- Fig. S1–S2: Frequencies of zeolite frameworks in the ZeoSyn dataset.
- Fig. S3–S4: Distributions of reaction conditions in the ZeoSyn dataset.
- Fig. S5: Frequencies of competing phases in the ZeoSyn dataset.
- Fig. S6: Confusion matrix of phase predictor model by pore sizes.
- Fig. S7: Structures of **RUT** and **IWW** frameworks.
- Fig. S8: CBU-level SHAP of selected small composite building units.
- Fig. S9: Framework-level SHAP of two competing phases (***BEA** and **BEC**).
- Fig. S10: CBU-level SHAP of all small composite building units.
- Fig. S11: CBU-level SHAP of all large composite building units.
- Fig. S12–S18: Framework-level SHAP of all zeolite frameworks in the ZeoSyn dataset.

Tables

- Table S1: OSDA physicochemical descriptors.
- Table S2: Frequencies of intergrowths in the ZeoSyn dataset.

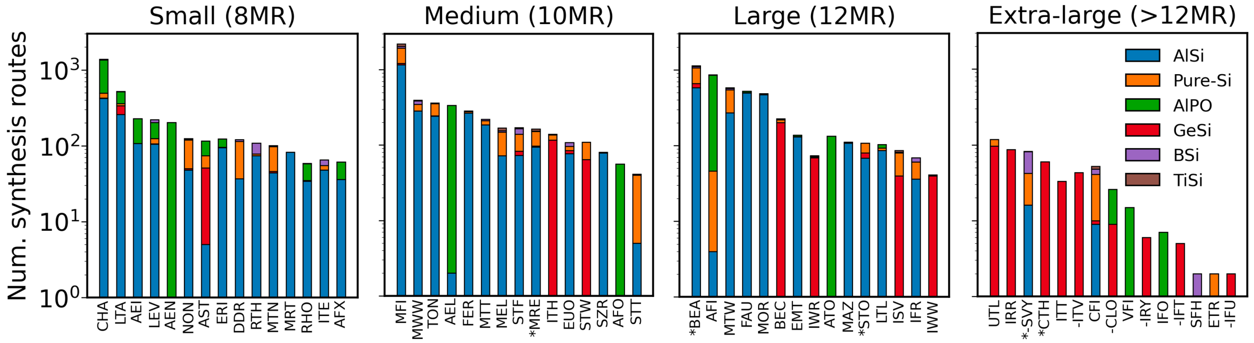

Figure S1: Number of synthetic routes for small, medium, large and extra-large pore frameworks in the dataset. Each framework is further broken down into its constituent zeotypes by color.

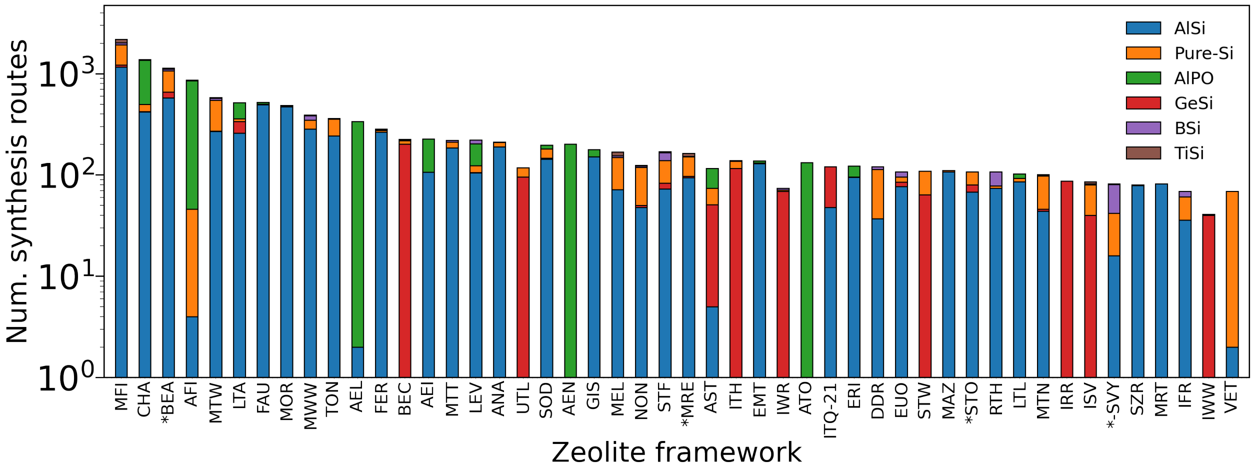

Figure S2: Number of synthesis routes of the 50 most frequent frameworks in the dataset.

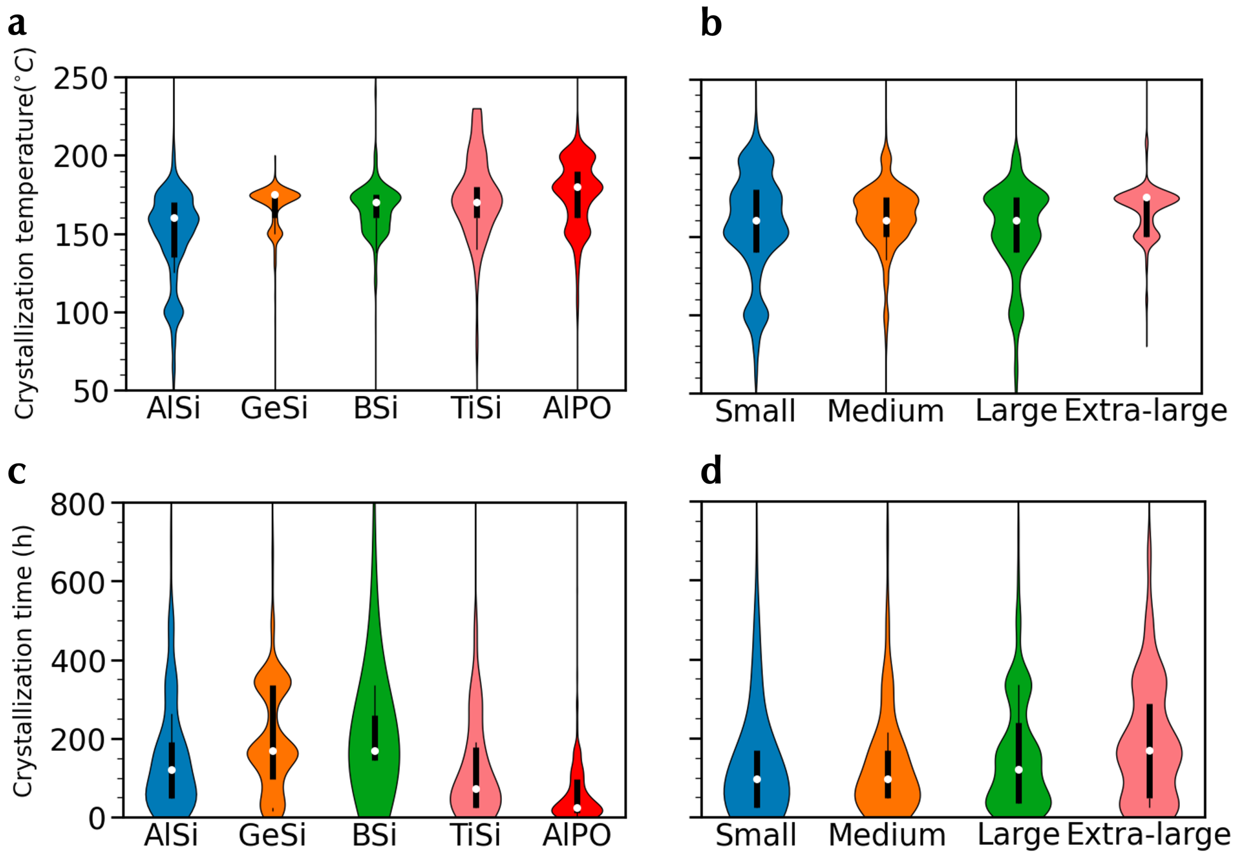

Figure S3: Distributions of reaction conditions in the dataset: crystallization temperatures (a/b) and times (c/d) of different zeotypes and pore-sizes in the dataset.

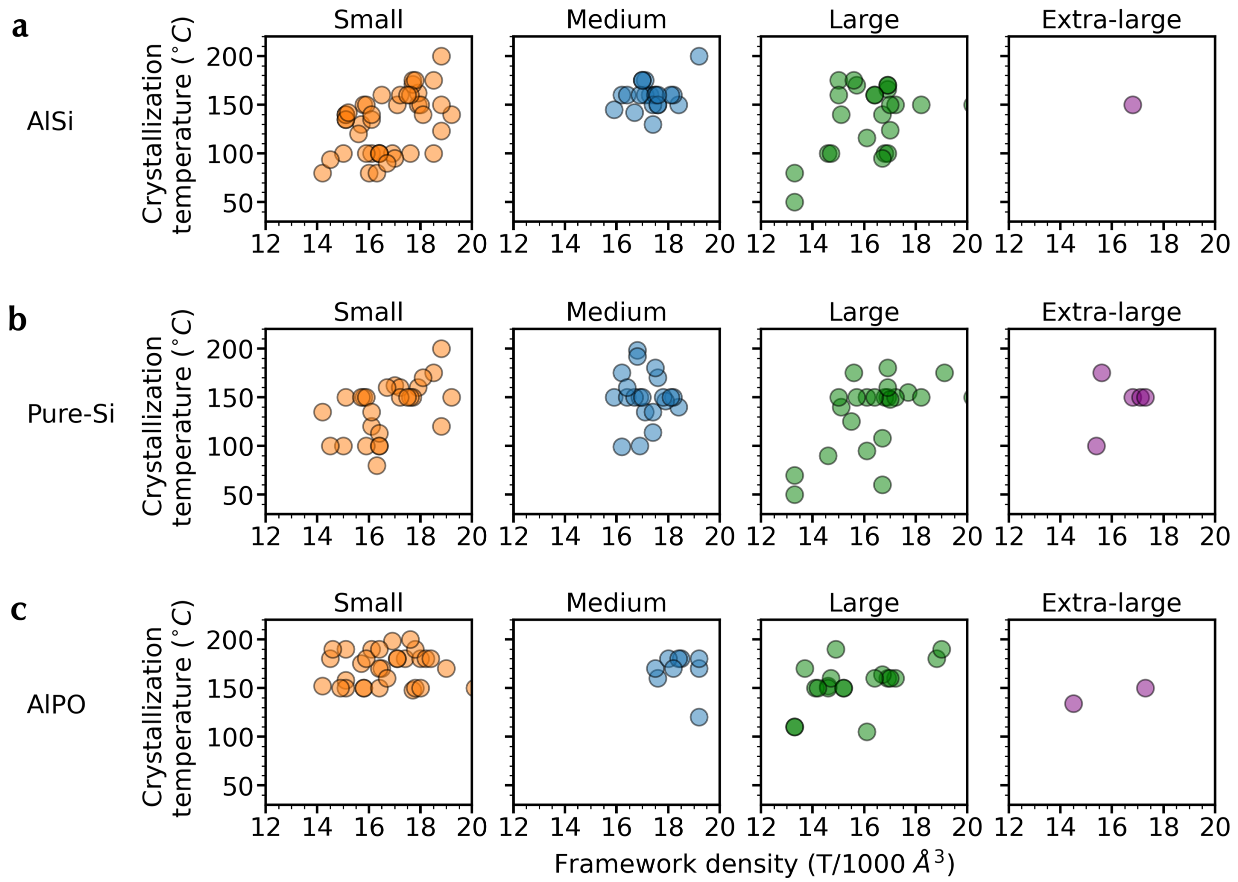

Figure S4: Relationship between crystallization temperature vs. framework density of zeolite for (a) aluminosilicate (b) pure-Si and (c) aluminophosphate frameworks across different pore sizes. Since multiple synthesis routes exist for a single framework type, for each framework we plot the crystallization temperature that corresponds to the 20th percentile for that framework in the dataset.

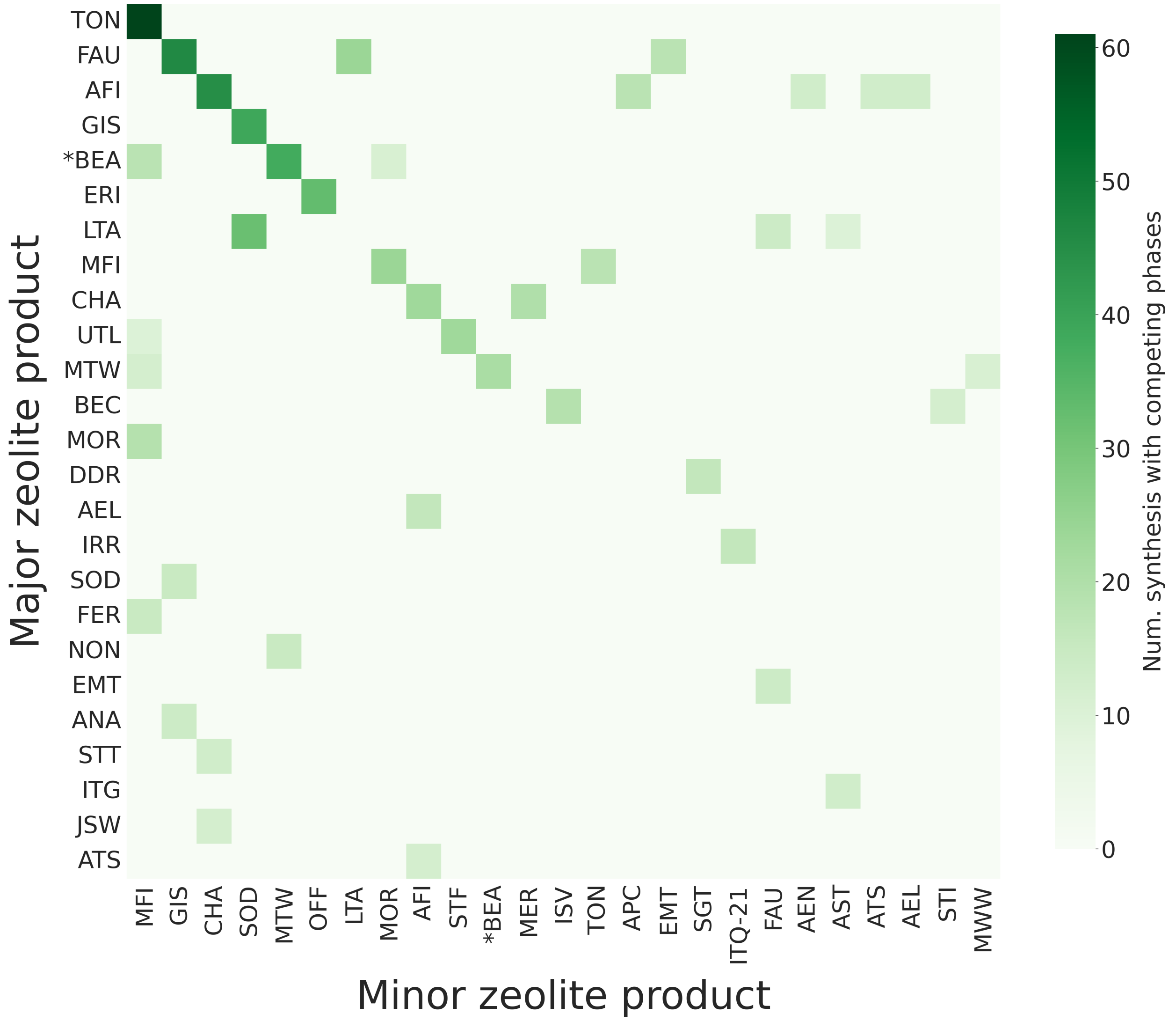

Figure S5: Number of synthesis routes with competing phases. y-axis is the major zeolite product, x-axis is the minor zeolite product.

Table S1: Physicochemical descriptors of OSDAs.

OSDA descriptor	Description
Asphericity	An anisometry descriptor for the deviation from the spherical shape
Axis 1	Two-dimensional (2D) shape descriptors of molecule calculated by projecting the atomic coordinates into a 2D space based on a principal component analysis (PCA) of the positions. The range of the distribution of points in each principal component is reported as the axis of the conformer. Axis 1 is reported as the larger axis, whereas Axis 2 is the smaller axis
Axis 2	See above
Charge	Formal charge of molecule
SASA	Solvent-accessible surface area (SASA) is the surface area of a molecule that is accessible to a solvent
Molecular weight	Molecular mass of molecule
NPR 1	Normalized principal moments ratio (I1/I3) where I is principal moment of inertia
NPR 2	Normalized principal moments ratio (I2/I3) where I is principal moment of inertia
Rotatable bonds	Number of rotatable bonds in the molecule. A measure of molecular flexibility.
PMI 1	Principal moments of inertia (PMI) are physical quantities related to the rotational dynamics of a molecule $$\mathrm{I} = \sum_{i=1}^{\mathrm{A}} m_i \cdot r_i^2 \quad (3)$$ where A is the number of atoms, and m_i is the atomic mass and r_i is the perpendicular distance from the chosen axis of the ith atom of the molecule
PMI 2	See above
PMI 3	See above
Sphericity	Sphericity index of molecule. A measure of how closely the shape of an object resembles that of a perfect sphere
Volume	Molecular volume calculated by using a grid-encoding of the molecular shape using a grid spacing of 0.2 Å and 2.0 Å of margin for the boxes

Table S2: Number of synthesis routes of intergrowths.

Intergrowth	Number of synthesis routes
ISV/BEC	167
ERI/OFF	74
MFI/MEL	47
TON/MTT	43
FAU/EMT	7
AFX/CHA	6
*BEA/BEC	5
CHA/AEI	7
STF/SFF	2
RUT/RTH	2
MEL/ZSM-55	1
MOR/MFI	1

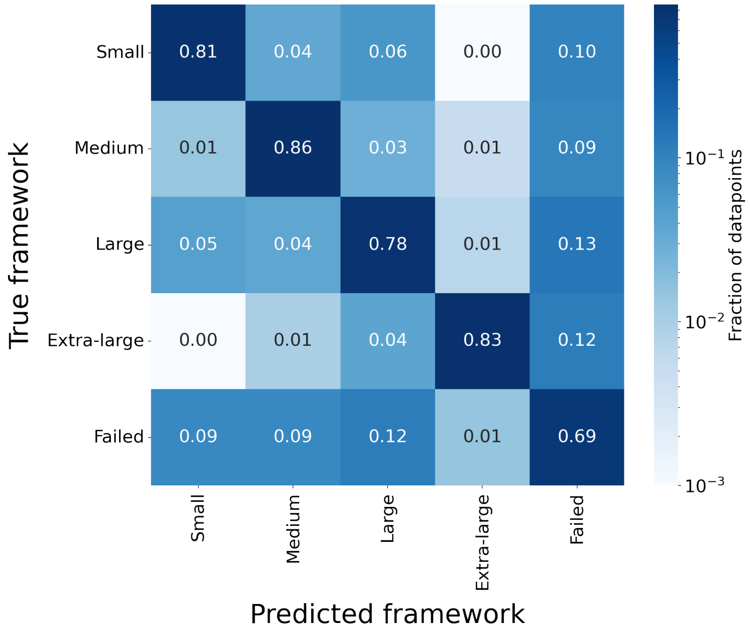

Figure S6: Normalized confusion matrix of phase predictor model. Here, we aggregate frameworks according to small, medium, large and extra-large pore frameworks. "Failed" refers to amorphous/dense phases.

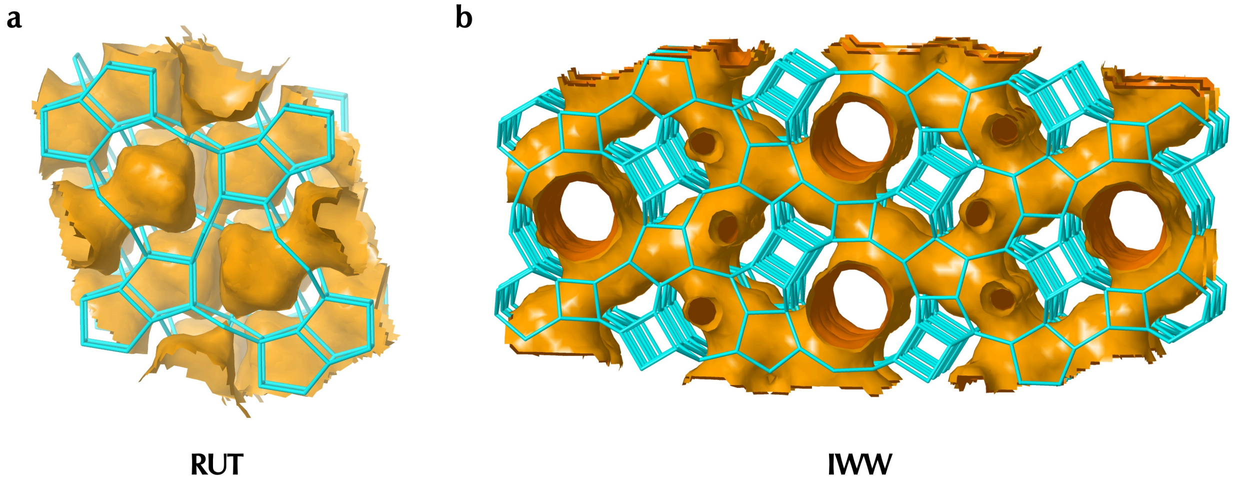

Figure S7: The **(a)** spherical cavities in **RUT** **(b)** long channels in **IWW** explain the difference in OSDA sphericity favoring the two frameworks: **IWW** is favored by spherical OSDAs while **RUT** is favored by longer OSDAs.

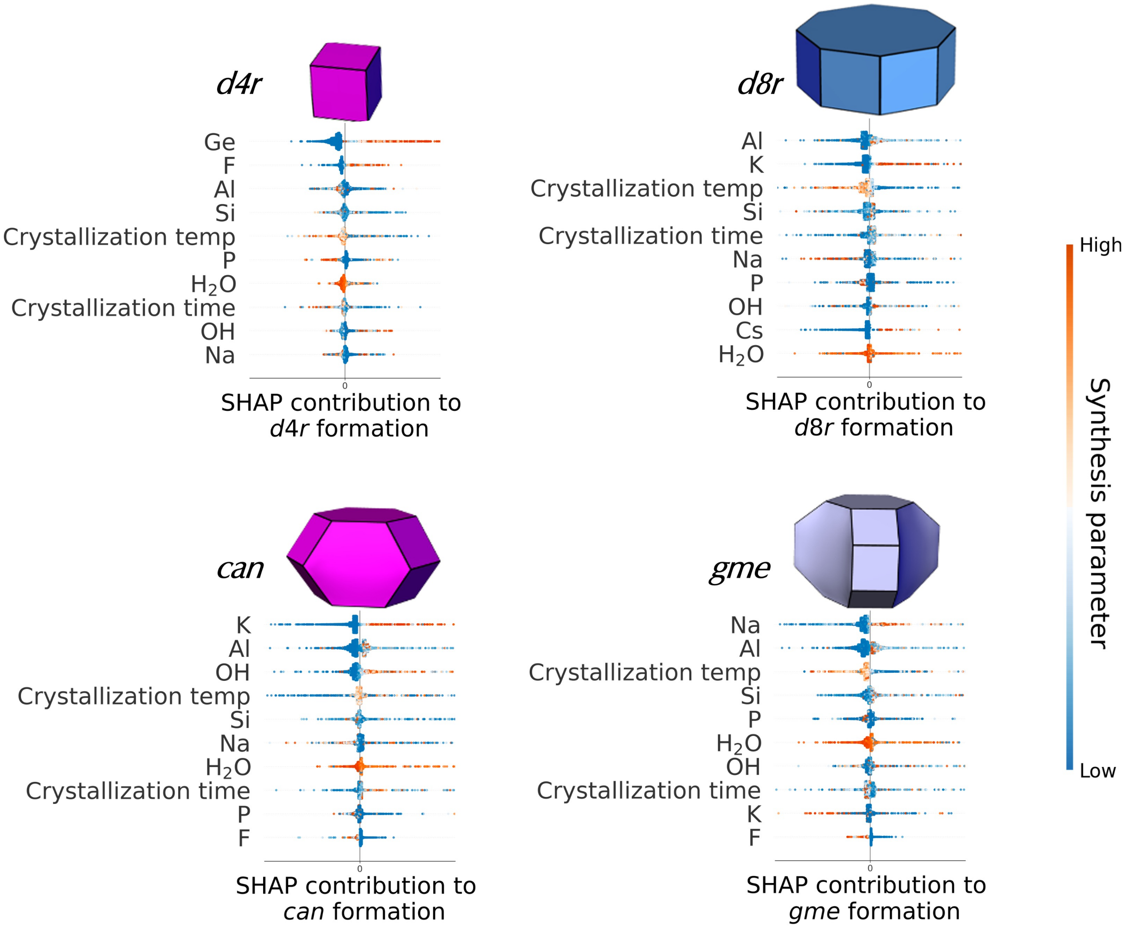

Figure S8: CBU-level SHAP analysis of small CBUs showing top 10 most important inorganic parameters (y-axis) contributing to their formation.

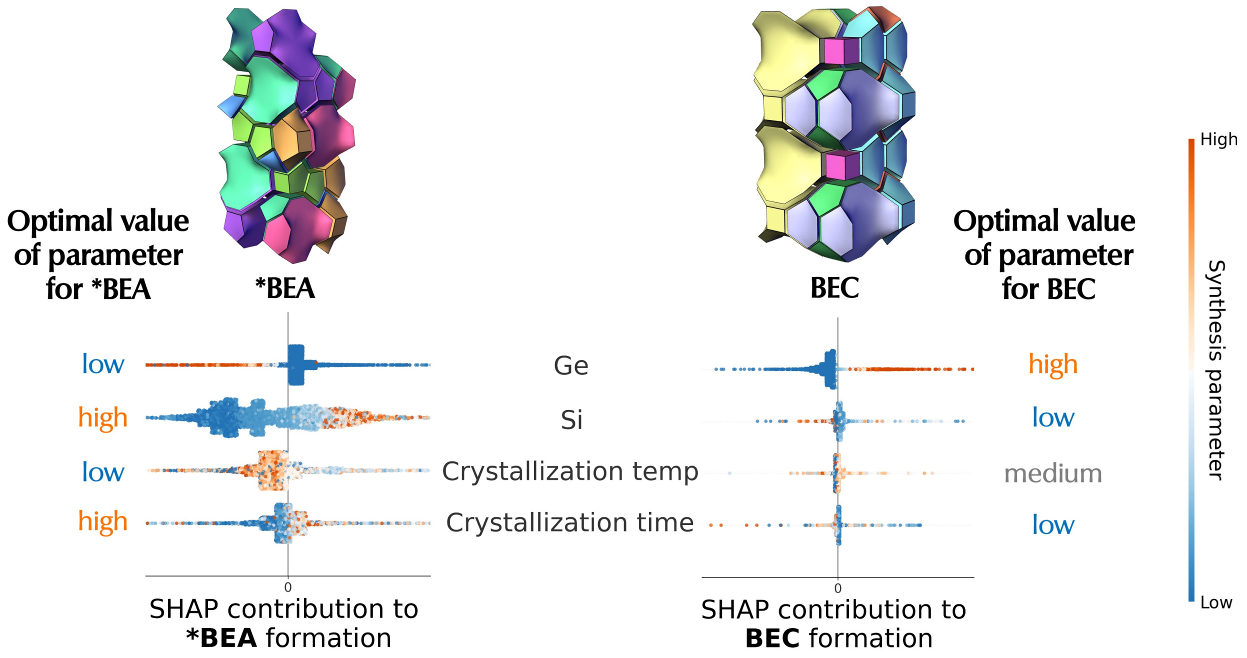

Figure S9: Application of framework-level SHAP on competing phases (***BEA** and **BEC**). The left- and right-most columns describe the optimal value of OSDA parameter for maximizing formation probability of ***BEA** and **BEC**, respectively.

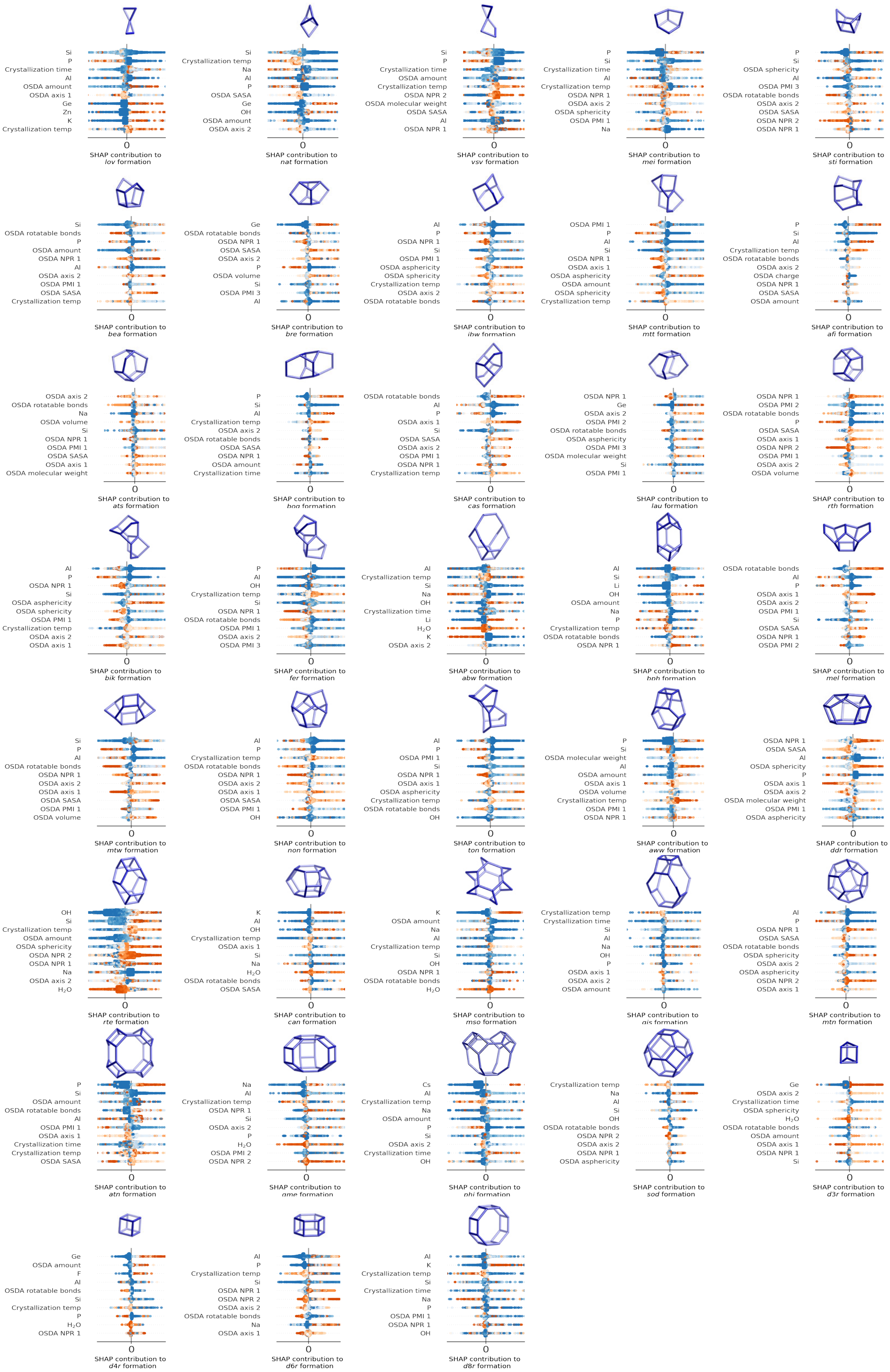

Figure S10: CBU-level SHAP of all small CBUs on IZA.

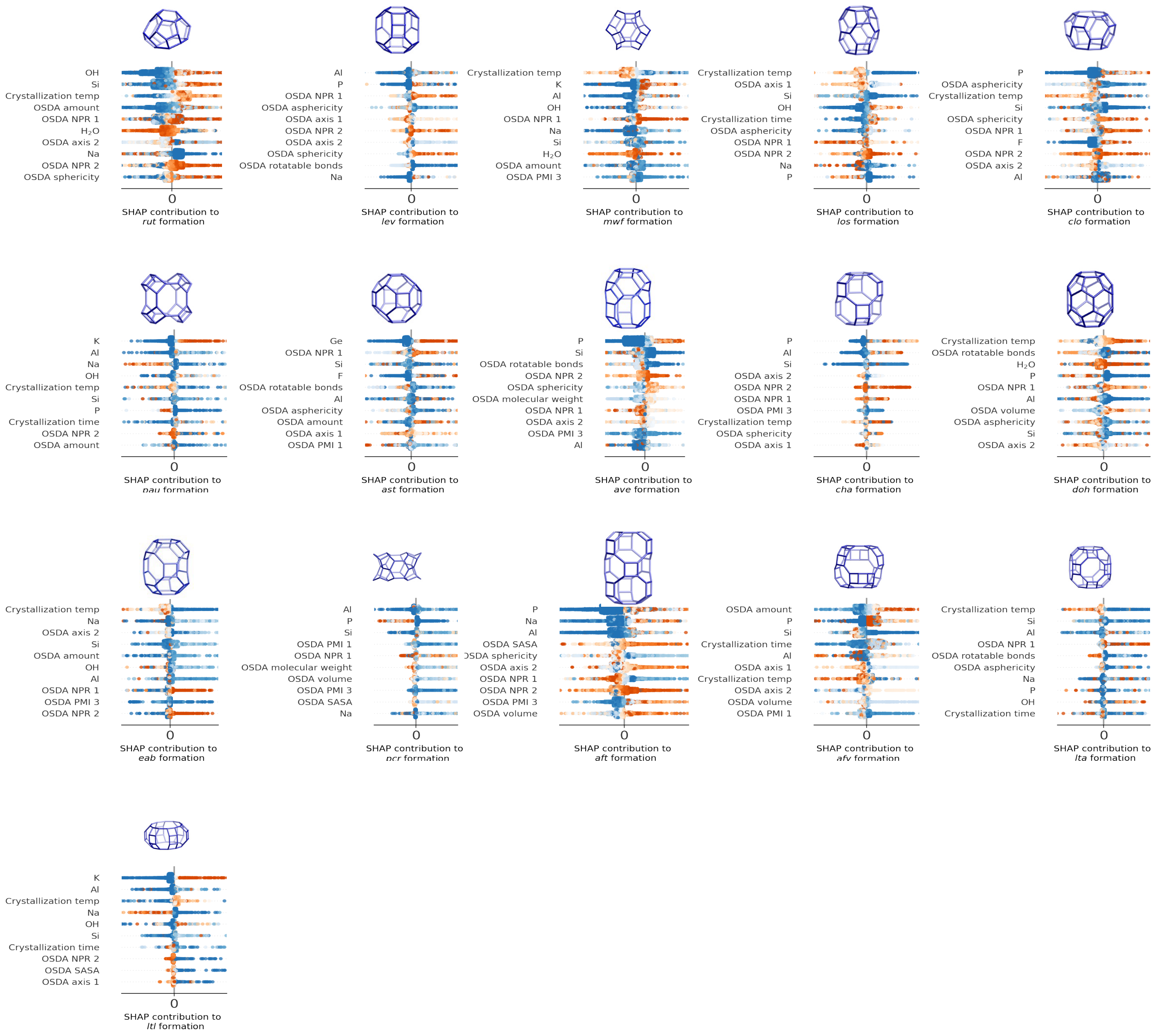

Figure S11: CBU-level SHAP of all large CBUs on IZA.

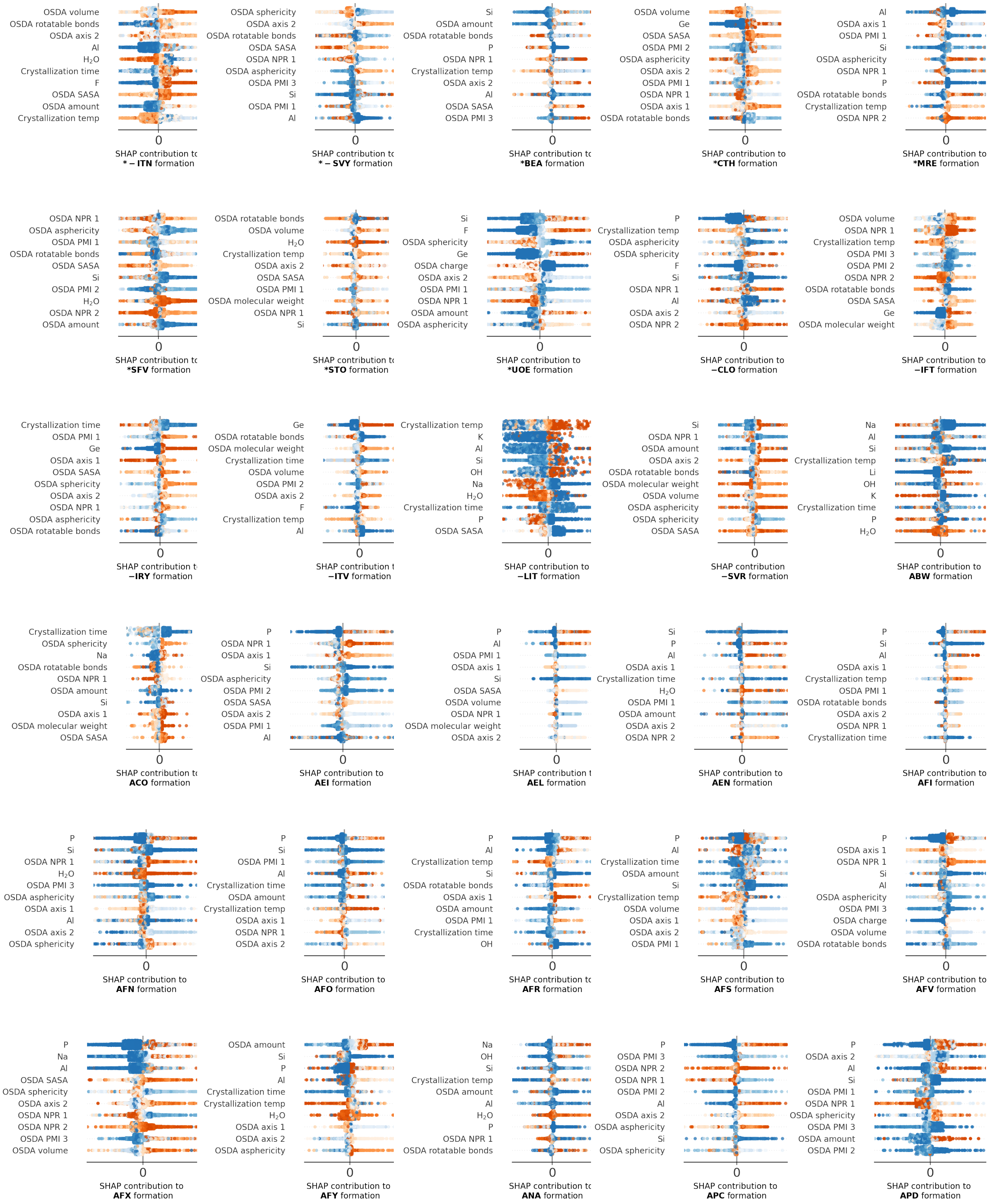

Figure S12: Framework-level SHAP of zeolite topologies (disordered and interrupted frameworks), and topologies with IZA code starting with A.

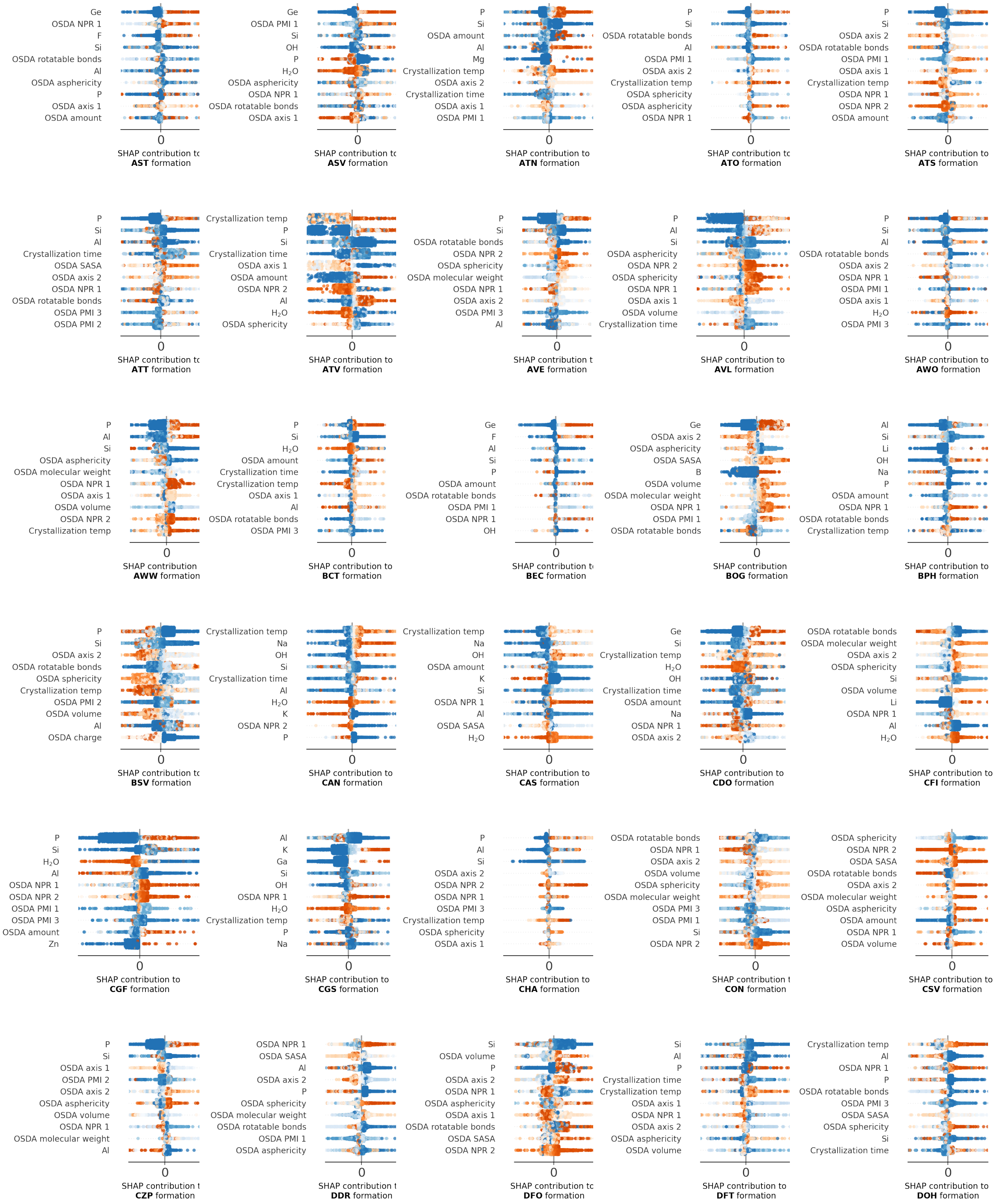

Figure S13: Framework-level SHAP of zeolite topologies with IZA code starting with A-D.

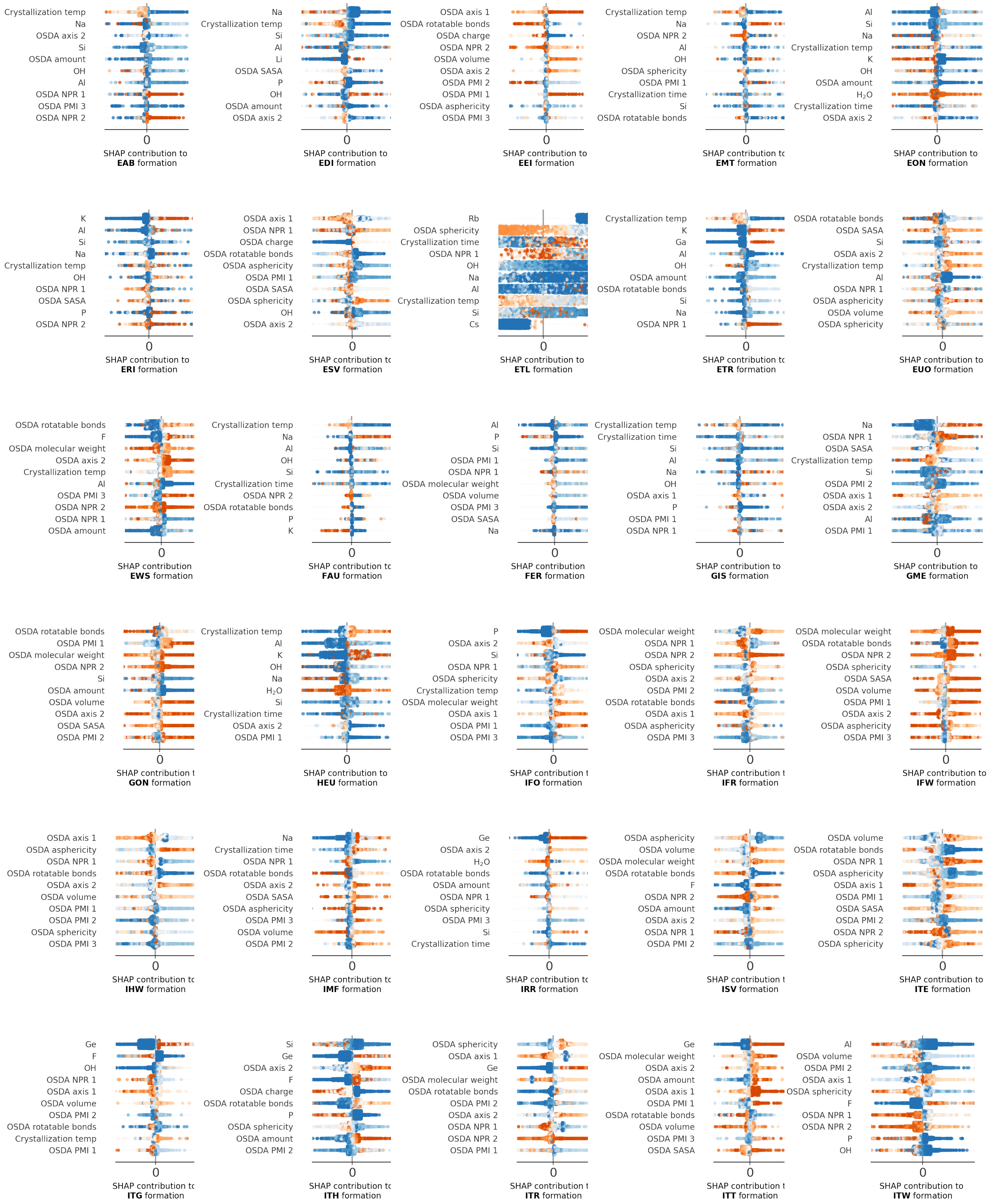

Figure S14: Framework-level SHAP of zeolite topologies with IZA code starting with E-I.

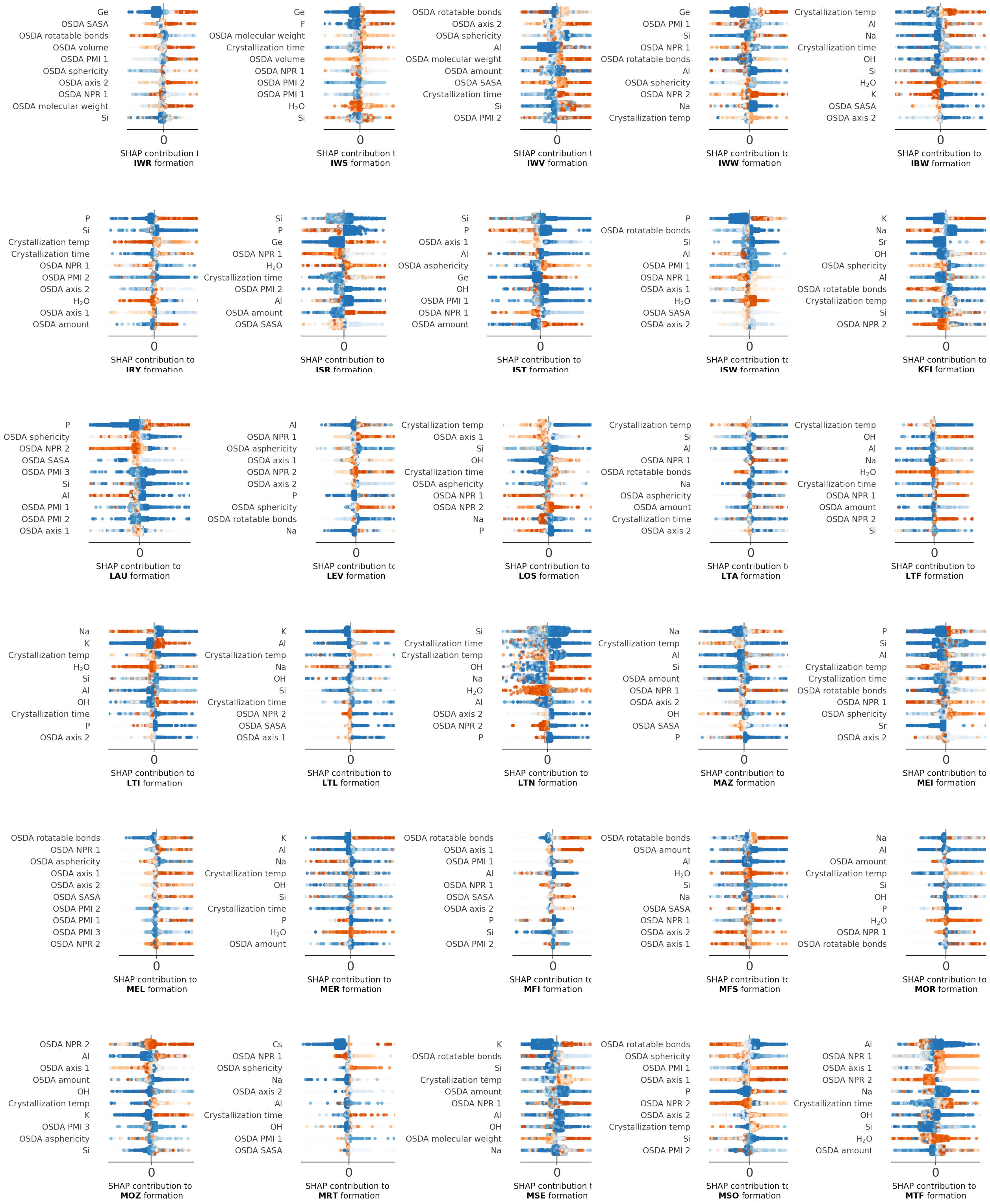

Figure S15: Framework-level SHAP of zeolite topologies with IZA code starting with I-M.

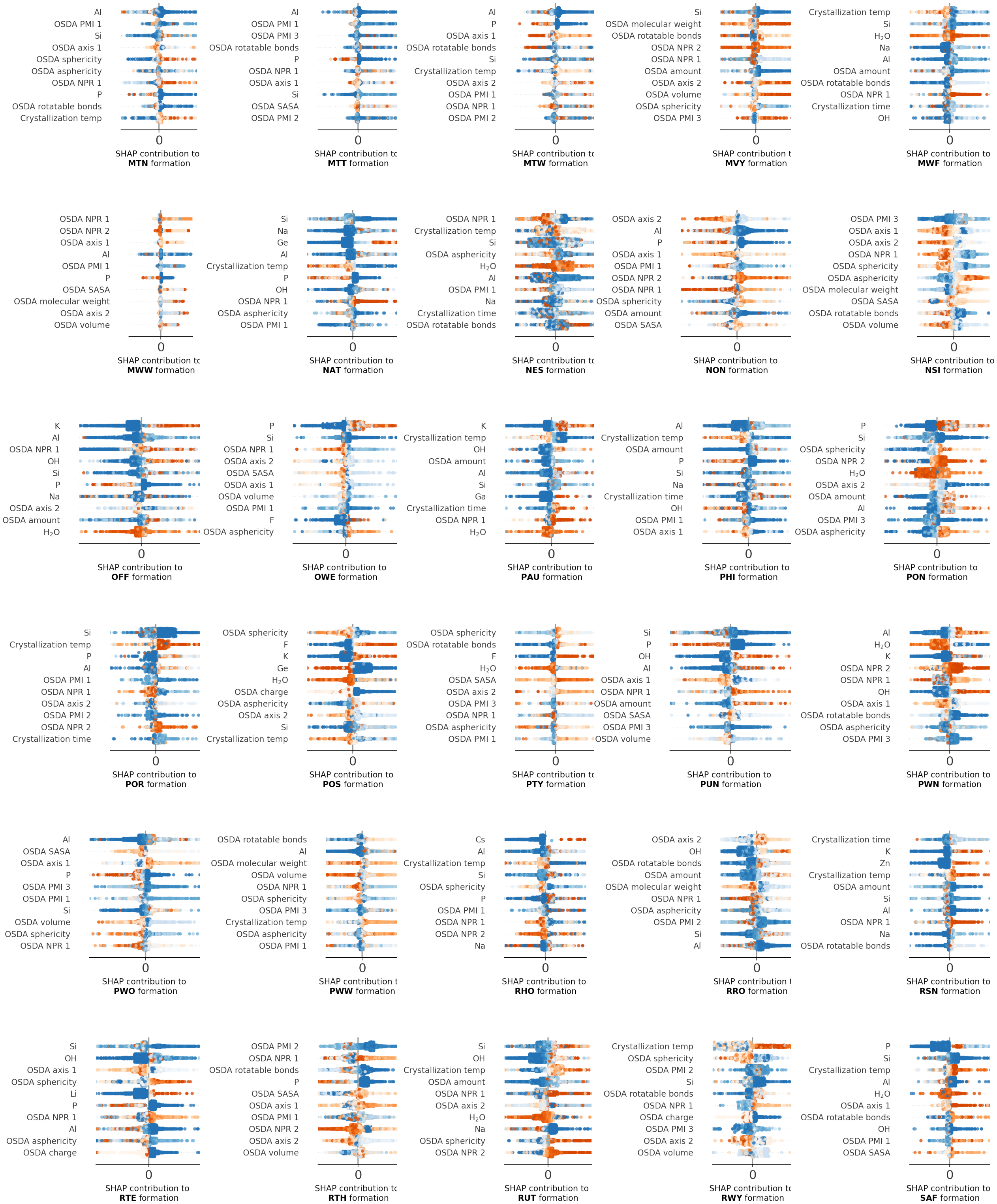

Figure S16: Framework-level SHAP of zeolite topologies with IZA code starting with M-S.

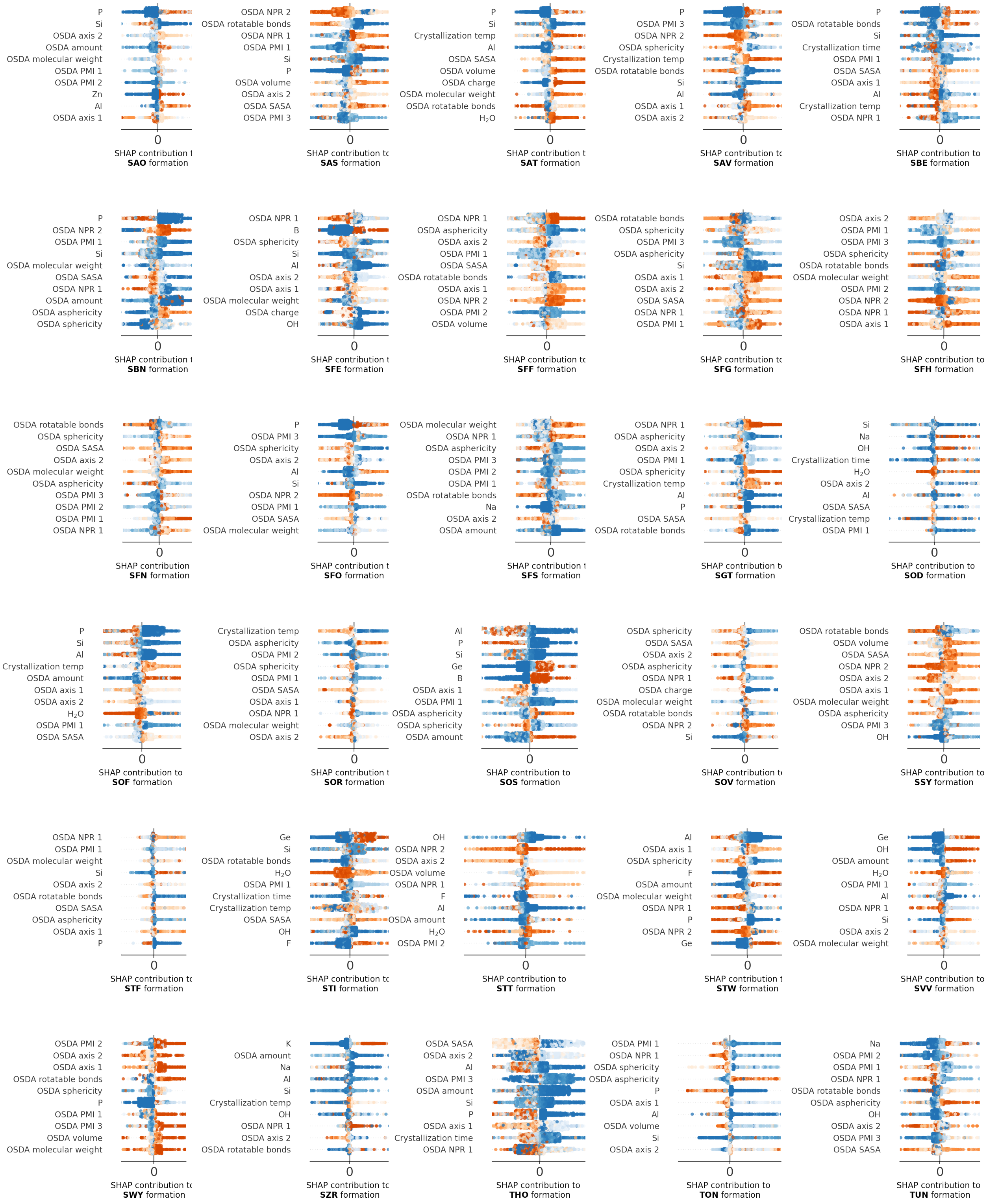

Figure S17: Framework-level SHAP of zeolite topologies with IZA code starting with S-T.

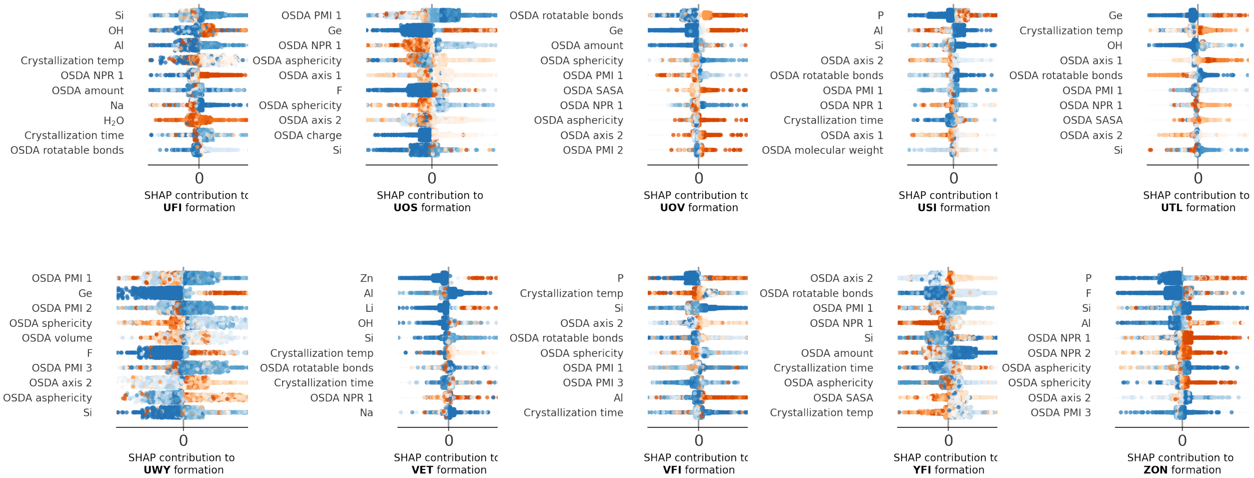

Figure S18: Framework-level SHAP of zeolite topologies with IZA code starting with U-Z.
